# Exploring the performance of ChatGPT on acute pancreatitis-related questions

**DOI:** 10.1186/s12967-024-05302-8

**Published:** 2024-06-01

**Authors:** Ren-Chun Du, Xing Liu, Yong-Kang Lai, Yu-Xin Hu, Hao Deng, Hui-Qiao Zhou, Nong-Hua Lu, Yin Zhu, Yi Hu

**Affiliations:** 1 Department of Gastroenterology, Digestive Disease Hospital, The First Affiliated Hospital of Nanchang University, 17 Yong Waizheng Street, Donghu District, Nanchang, 330006 Jiangxi Province China; 2 Department of Gastroenterology, Ganzhou People’s Hospital Affiliated to Nanchang University, Ganzhou, China; 3 Department of Gastroenterology, Shanghai Changhai Hospital, Naval Medical University, Shanghai, China; 4 School of Math and Computer, Nanchang University, Nanchang, Jiangxi Province China; 5 Faculty of Medicine, Macau University of Science and Technology, Macau, China; 6 Department of Surgery, The Chinese University of Hong Kong, Shatin NT, Hong Kong, China

Letter to the editor:

Acute pancreatitis (AP) is a serious gastrointestinal disease with an incidence rate of approximately 34 cases per 100,000 individuals annually, the overall burden of AP remains high with the aging population [1]. There is a notable trend among the public to acknowledge AP-related information to improve awareness.

Artificial intelligence (AI) is a large language model providing updated and useful information. The Chat Generative Pre-trained Transformer (ChatGPT, https://openai.com), developed by OpenAI and launched on November 30, 2022, stands out in this field. Various studies have explored its utility in responding to medical questions. This study aims to evaluate and compare the capabilities of ChatGPT-3.5 and ChatGPT-4.0 in answering test questions about AP, employing both subjective and objective metrics.

## Methods

As shown in Table S1, we conducted our study using 18 subjective test questions derived from the Atlanta AP classification consensus and the American Gastroenterological Association (AGA) guidelines (Strength of recommendation: Strong) [2–4]. Additionally, we selected 73 objective questions with the highest number of tested times from the Chinese professional physician test database, categorizing them into four subfields (Table S2). These questions were submitted to ChatGPT in two separate sessions on February 1, 2024, and February 8, 2024, respectively. Two independent reviewers evaluated the subjective questions using a 5-point Likert Scale. Any discordance was resolved by the third author. The flowchart of overall study design is presented in Figure S1. The response accuracy was analyzed using the Chi-squared and Mann–Whitney U tests, with a *P*-value of < 0.05 indicating statistical significance.

## Results

As shown in Table 1, ChatGPT-3.5 correctly answered 80% of subjective questions, while ChatGPT-4.0 achieved an accuracy rate of 94%. For objective questions, ChatGPT-4.0 outperformed ChatGPT-3.5 with a 78.1% accuracy rate compared to 68.5% (*P* = 0.01) (Figure S2A). Across all questions tested in the study, the concordance rate between ChatGPT-3.5 and ChatGPT-4.0 was 80.8% and 83.6% (Figure S2B), respectively, with the mean number of words per response being 218.5 for ChatGPT-3.5 and 246.0 for ChatGPT-4.0 (Table 1). Notably, correct answers showed higher concordance rates than incorrect ones across both versions of ChatGPT (95.7% and 91.1% vs. 55.6% and 58.8%) (Table 2). Notably, both ChatGPT-3.5 and ChatGPT-4.0 demonstrated high accuracy rates, particularly in the etiology category.

**Table 1:** Quality indicators (scientific adequacy) for answers from ChatGPT version 3.5 and 4.0

Common questions	Sources of answers	Words		Grades “The answers are scientifically adequate”	
		Response 1	Response 2	Mean	*P*-value
All (Mean)	ChatGPT 3.5	255	237	4	< 0.01
	ChatGPT 4.0	202	235	4.7	
Basic knowledge					
What are of types of acute pancreatitis?	ChatGPT 3.5	138	167	5	1
	ChatGPT 4.0	165	164	5	
What is the difference between mild, moderate and severe acute pancreatitis?	ChatGPT 3.5	218	225	5	1
	ChatGPT 4.0	316	284	5	
What is the identification of two distinct phases of acute pancreatitis?	ChatGPT 3.5	213	172	4	0.029
	ChatGPT 4.0	246	175	5	
Diagnosis					
What is the diagnosis criteria of acute pancreatitis?	ChatGPT 3.5	169	185	4	0.029
	ChatGPT 4.0	123	146	5	
What are the common symptoms of acute pancreatitis?	ChatGPT 3.5	190	156	5	1
	ChatGPT 4.0	183	186	5	
What is the signs of acute pancreatitis systemic inflammatory response syndrome (SIRS)?	ChatGPT 3.5	191	165	3 4.5	0.029
	ChatGPT 4.0	104	166		
Treatment					
What is the initial management of acute pancreatitis?	ChatGPT 3.5	317	276	4	0.686
	ChatGPT 4.0	263	337	4.25	
When should patients with acute pancreatitis combined with acute cholangitis receive Endoscopic Retrograde Cholangiopancreatography (ERCP)?	ChatGPT 3.5	286	299	3	0.343
	ChatGPT 4.0	137	160	4	
What should be done for an acute pancreatitis extrapancreatic infection, such as cholangitis, catheter-acquired infections, bacteremia, urinary tract infections, and pneumonia?	ChatGPT 3.5	319	269	4	0.686
	ChatGPT 4.0	331	339	4.25	
Should prophylactic antibiotics be routinely used in patients with severe acute pancreatitis?	ChatGPT 3.5	306	240	3.5	0.029
	ChatGPT 4.0	139	203	5	
Should patients with sterile necrosis use antibiotics to prevent the development of infected necrosis?	ChatGPT 3.5	322	225	2	0.029
	ChatGPT 4.0	165	189	5	
Should patients with mild acute pancreatitis found to have gallstones in the gallbladder receive cholecystectomy before discharge?	ChatGPT 3.5	255	286	3	0.029
	ChatGPT 4.0	190	204	4	
Does the presence of asymptomatic pseudocysts and pancreatic and / or extrapancreatic necrosis require intervention?	ChatGPT 3.5	284	261	3.5	0.343
	ChatGPT 4.0	201	369	4	
Is maintaining enteral nutrition thought to be helpful for patients with acute pancreatitis?	ChatGPT 3.5	318	300	5	1
	ChatGPT 4.0	143	221	5	
Prevention					
What are the most common causes of acute pancreatitis?	ChatGPT 3.5	194	221	5	1
	ChatGPT 4.0	171	250	5	
What are the well-studied interventions for patients undergoing a therapeutic ERCP to decrease the risk of post-ERCP pancreatitis, especially severe disease?	ChatGPT 3.5	331	322	4	0.343
	ChatGPT 4.0	220	342	4.5	
Others					
What is the definition of idiopathic acute pancreatitis?	ChatGPT 3.5	174	208	5	1
	ChatGPT 4.0	190	170	5	
Do we need more evidence to optimize the management of acute pancreatitis?	ChatGPT 3.5	371	286	4	1
	ChatGPT 4.0	342	331	4

**Table 2:** Performance of ChatGPT 3.5, ChatGPT 4.0 and medical college examinees on acute pancreatitis test questions and by different subfields

Test questions by subfields	ChatGPT 3.5		ChatGPT 4.0		Examinees
	Correct	Incorrect	Correct	Incorrect	
All test questions, No.	73		73		
1st run, No. (%)	46 (63.0)	27 (37.0)	56 (76.7)	17 (23.3)	
2nd run, No. (%)	56 (76.7)	17 (23.3)	58 (79.5)	15 (20.5)	
Concordance between 2 runs, No. (%)	44 (95.7)	15 (55.6)	51 (91.1)	10 (58.8)	
Total concordance, No. (%)	59 (80.8)		61 (83.6)		
Total accuracy (%)	68.5		78.1		72.4
Diagnosis, No.	43		43		
1st run, No. (%)	26 (60.5)	17 (39.5)	34 (79.1)	9 (20.9)	
2nd run, No. (%)	32 (74.4)	11 (25.6)	35 (81.4)	8 (18.6)	
Concordance between 2 runs, No. (%)	26 (100)	11 (64.7)	31 (91.2)	5 (66.7)	
Total concordance, No. (%)	37 (86.0)		36 (83.7)		
Total accuracy (%)	65.1		80.2		75.3
Clinical feature, No.	9		9		
1st run, No. (%)	4	5	7	2	
2nd run, No. (%)	6	3	6	3	
Concordance between 2 runs, No. (%)	3 (75.0)	2 (40.0)	6 (85.7)	2 (100.0)	
Total concordance, No. (%)	5 (55.6)		8 (88.9)		
Total accuracy (%)	55.6		72.2		60.0
Treatment, No.	12		12		
1st run, No. (%)	7 (58.3)	5 (41.7)	8 (58.3)	4 (41.7)	
2nd run, No. (%)	9 (75.0)	3 (25.0)	9 (75.0)	3 (25.0)	
Concordance between 2 runs, No. (%)	6 (85.7)	2 (40.0)	7 (87.5)	2 (50.0)	
Total concordance, No. (%)	8 (66.7)		9 (75.0)		
Total accuracy (%)	66.7		70.8		69.1
Etiology, No.	9		9		
1st run, No. (%)	9 (100.0)	0	7 (77.8)	2 (22.2)	
2nd run, No. (%)	9 (100.0)	0	8 (88.9)	1 (11.1)	
Concordance between 2 runs, No. (%)	9 (100.0)	0	7 (100.0)	1 (50.0)	
Total concordance, No. (%)	9 (100)		9 (100)		
Total accuracy (%)	100		83.3		75.2

## Discussion

Our findings indicate that ChatGPT-4.0 outperformed ChatGPT-3.5 in answering both subjective and objective test questions related to AP, demonstrating a superior total accuracy. The accuracy of both ChatGPT-3.5 and the examinees in responding to clinical feature test questions was generally low, which suggests that clinical features associated with AP are complex, often involving numerous complications, which makes identifying the optimal solution challenging.

In addressing subjective questions, ChatGPT tends to provide a range of answers, mixing relevant with irrelevant information, making it challenging to discern the most accurate answer for healthcare professionals and patients. This discrepancy highlights the lower accuracy rate for objective choice questions compared to subjective ones. However, ChatGPT-4.0 showed improvements in providing more precise, concise, and focused answers.

Although ChatGPT answered most subjective questions correctly, the standard answers were conducted based on early guideline evidence. A significant limitation of artificial intelligence is its inability to update information in real-time. Recent randomized controlled trials focusing on AP have presented evidence that questions existing management strategies, such as the use of antibiotics, fluid resuscitation, the handling of infected necrosis, and the early application of ERCP [5]. It is imperative to reevaluate the current management guidelines to ensure they reflect the latest evidence.

This study has several limitations. Firstly, although we conducted two separate evaluations, the results might be influenced by the timing of the assessments of ChatGPT. Secondly, we did not incorporate patient perspectives, which are crucial as they are the ultimate recipients of AP-related information. Thirdly, the study participants were medical students, and we lacked data from practicing doctors.

In conclusion, ChatGPT-4.0 exhibited superior performance compared to ChatGPT-3.5. However, both versions of ChatGPT tended to provide broad and generalized answers across various topics and aspects, rather than offering optimal solutions. Therefore, ChatGPT excels at addressing subjective questions and offering a wide range of options, but it is not suitable for providing optimal management strategies, and cannot adjust treatment plans based on the latest evidence, where enhancements in training are required.

### Supplementary Information

Supplementary Material 1: Figure S1: Flowchart of overall study design

Supplementary Material 2: Figure S2: Comparison of accuracy of ChatGPT-4.0, ChatGPT-3.5 and examinees on acute pancreatitis test objective questions (A); Comparison of concordance of ChatGPT-4.0, ChatGPT-3.5 on acute pancreatitis test objective questions (B)

Supplementary Material 3.

Supplementary Material 4.

## Data Availability

Not applicable.
