# Supplementary figures and images for: Exploring the performance of ChatGPT on acute pancreatitis-related questions
Source: J Transl Med. 2024 Jun 1;22:527. doi: 10.1186/s12967-024-05302-8 (PMC11143553; doi:10.1186/s12967-024-05302-8)

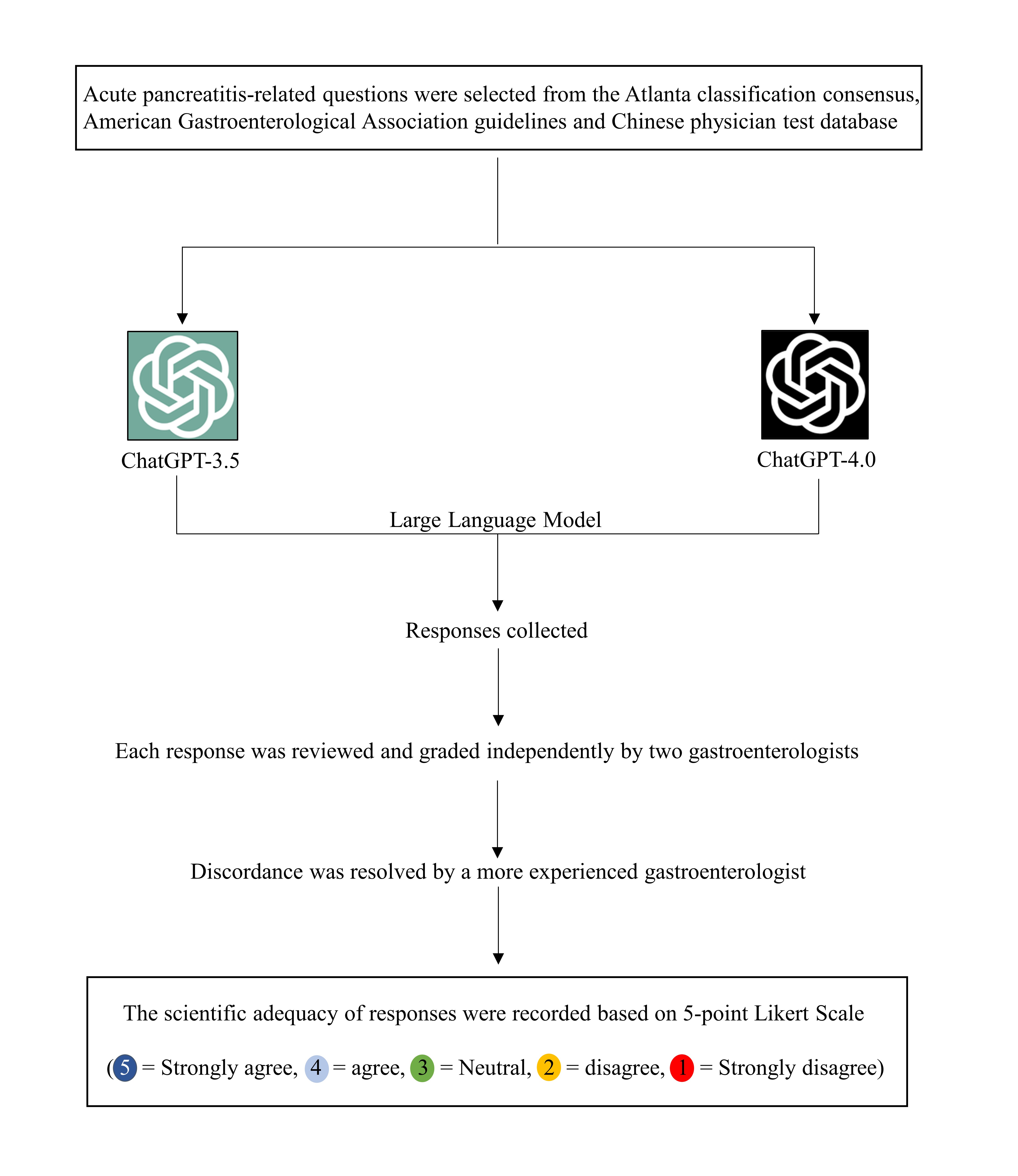

Supplement: Supplementary file 1 — Supplementary Material 1: Figure S1: Flowchart of overall study design [file 12967_2024_5302_MOESM1_ESM.jpg]

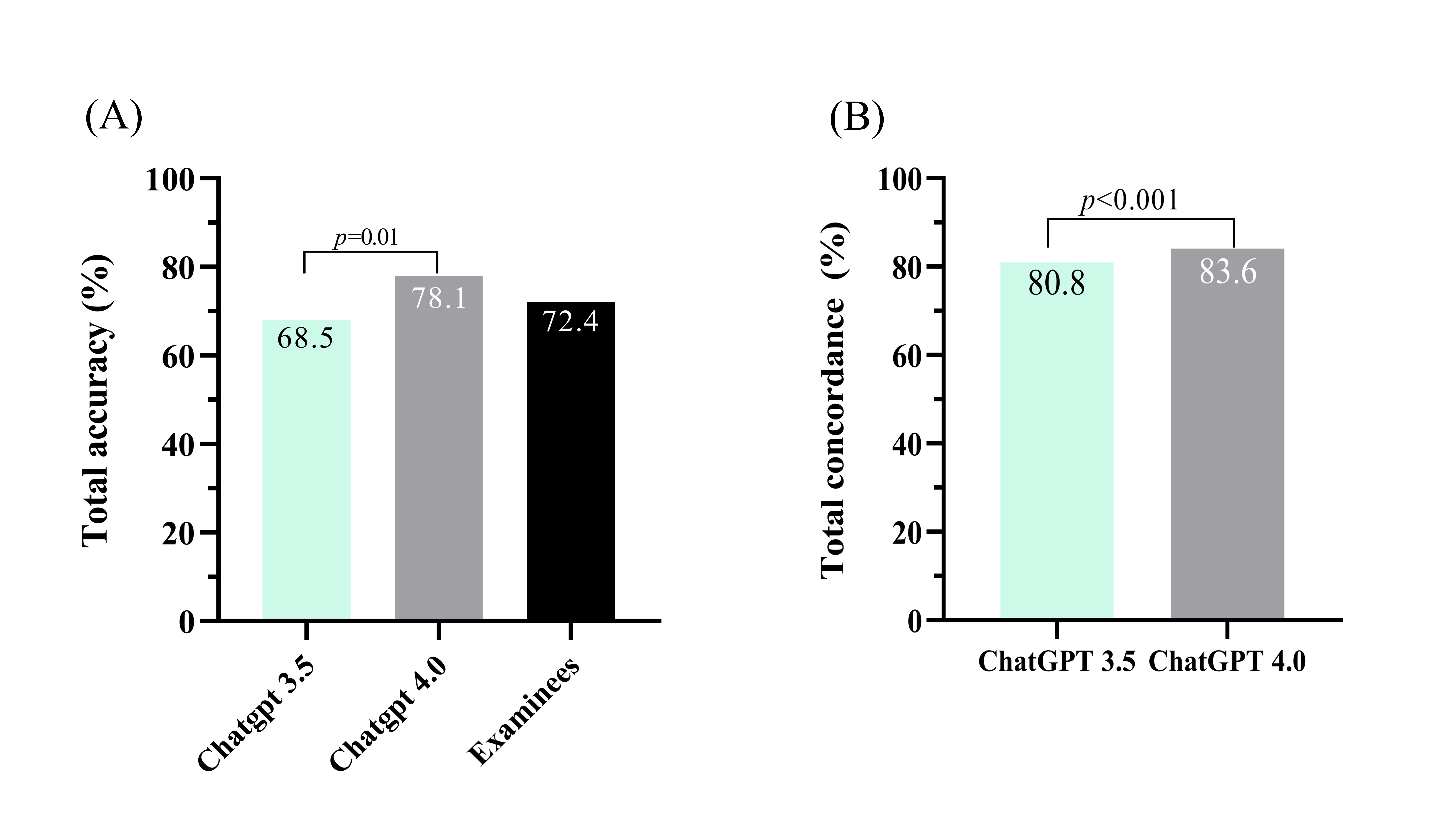

Supplement: Supplementary file 2 — Supplementary Material 2: Figure S2: Comparison of accuracy of ChatGPT-4.0, ChatGPT-3.5 and examinees on acute pancreatitis test objective questions (A); Comparison of concordance of ChatGPT-4.0, ChatGPT-3.5 on acute pancreatitis test objective questions (B) [file 12967_2024_5302_MOESM2_ESM.jpg]
