# Supplementary material for: Exploring the performance of ChatGPT on acute pancreatitis-related questions
Source: J Transl Med. 2024 Jun 1;22:527. doi: 10.1186/s12967-024-05302-8 (PMC11143553; doi:10.1186/s12967-024-05302-8)
Supplement: Supplementary file 3 — Supplementary Material 3. [file 12967_2024_5302_MOESM3_ESM.docx]

| **Table S1 Example answers from ChatGPT and standard answers from consensus.** | | | |
| --- | --- | --- | --- |
| Common Questions | Response from ChatGPT 3.5 | Response from ChatGPT 4.0 | Standard answers from consensus |
| **Basic knowledge** |  |  |  |
| What are of types of acute pancreatitis based on the extent of inflammation and tissue damage within the pancreas? | Response 1: Acute pancreatitis can be classified based on the extent of inflammation and tissue damage within the pancreas. The two main types are:  Interstitial Edematous Pancreatitis: This is the milder form of acute pancreatitis where inflammation is confined to the pancreatic interstitium (the spaces between cells). Edema, or swelling, occurs in the pancreas, but there is generally minimal tissue necrosis (death). Patients with interstitial edematous pancreatitis often recover with supportive care, which may include fasting, pain management, and hydration.  Necrotizing Pancreatitis: This is a more severe form of acute pancreatitis characterized by the presence of tissue necrosis (cell death) in addition to inflammation. Necrotizing pancreatitis can involve both the pancreas and the surrounding tissues. It is associated with a higher risk of complications, such as infection, and may require more aggressive interventions, including surgical procedures to remove necrotic tissue.  Response 2: Acute pancreatitis can be classified into different types based on the extent of inflammation and tissue damage within the pancreas. The most commonly used classification system is the Revised Atlanta Classification, which categorizes acute pancreatitis into two main types:  Interstitial Edematous Pancreatitis (Edematous or Mild Pancreatitis):  This type is characterized by inflammation and edema (swelling) of the pancreas without significant tissue necrosis (cell death).  The inflammatory process primarily involves the pancreatic interstitium (the supportive tissue between cells), and patients with this type generally have a better prognosis compared to the necrotizing type.  Necrotizing Pancreatitis: This type involves more severe inflammation, leading to necrosis (death) of pancreatic tissue.  Necrotizing pancreatitis can be further classified into two subtypes: a. Necrotizing Pancreatitis Without Infection: In this subtype, necrosis occurs, but there is no evidence of infection within the necrotic tissue. b. Necrotizing Pancreatitis With Infection: In this subtype, infection develops within the necrotic areas of the pancreas. Infected necrosis is associated with a higher risk of complications and increased mortality. | Response 1: Acute pancreatitis is traditionally classified based on the extent and nature of the inflammation and tissue damage within the pancreas. The two main types of acute pancreatitis are:  Interstitial Edematous Pancreatitis (IEP):  This is the most common form, accounting for about 80-90% of cases.  It involves inflammation and edema (swelling) within the pancreas but without extensive tissue death or necrosis.  The pancreas typically appears enlarged and edematous on imaging.  The condition usually resolves without severe complications, although it can be painful and may require hospitalization.  Necrotizing Pancreatitis (NP):  This type is more severe and less common, representing 10-20% of cases.  It involves the death of pancreatic tissue (necrosis), which can affect only the pancreatic tissue itself (pancreatic necrosis) or the pancreatic tissue and the surrounding fatty tissue (pancreatic and peripancreatic necrosis).  Necrosis can lead to more serious complications such as infection, abscess formation, or pseudocysts.  This type often requires longer hospital stays, and sometimes surgical intervention is needed to remove necrotic tissue or address complications.  Response 2: Acute pancreatitis is categorized based on the extent of inflammation and tissue damage into two main types:  Interstitial Edematous Pancreatitis (IEP):  This type is characterized by swelling and inflammation of the pancreatic tissue without tissue death (necrosis).  It is the less severe form of acute pancreatitis and is more common, accounting for 80-90% of cases.  The pancreas appears enlarged and there is increased interstitial fluid, but there's no substantial necrosis or destruction of pancreatic tissue.  Most patients with IEP recover without serious complications after receiving appropriate medical treatment.  Necrotizing Pancreatitis (NP):  This is the more severe form of acute pancreatitis and involves the death of pancreatic tissue, known as necrosis.  Necrosis can be confined to the pancreas (pancreatic necrosis) or it can also affect the tissues surrounding the pancreas (peripancreatic necrosis).  Approximately 10-20% of acute pancreatitis cases are necrotizing.  Necrotizing pancreatitis carries a higher risk of complications such as infection, pseudocyst formation, and systemic illness, and may require more aggressive treatment including surgical intervention. | Acute pancreatitis can be subdivided into two types: interstitial oedematous pancreatitis and necrotising pancreatitis. |
| What is the difference between mild, moderate and severe acute pancreatitis? | Response 1: The severity of acute pancreatitis is typically classified into three categories: mild, moderate, and severe. This classification is based on various clinical, laboratory, and imaging criteria. The severity of acute pancreatitis helps guide the management and treatment approach. The criteria for each category may vary slightly among different guidelines, but the following are general characteristics for each:  Mild Acute Pancreatitis: Patients with mild acute pancreatitis usually have no organ failure or local or systemic complications.  There is minimal or no pancreatic necrosis (death of pancreatic tissue).  Symptoms are typically self-limited, and the patient may recover with supportive care, such as pain management, hydration, and monitoring for potential complications.  Moderate Acute Pancreatitis: Patients with moderate acute pancreatitis may exhibit transient organ failure that resolves within 48 hours.  There may be local complications, such as fluid collections or necrosis, but they are not extensive.  These patients may require more intensive monitoring and supportive care, and in some cases, interventions to manage complications.  Severe Acute Pancreatitis: Severe acute pancreatitis is characterized by persistent organ failure that lasts for more than 48 hours.  Extensive pancreatic necrosis and/or other severe complications may be present.  Patients with severe acute pancreatitis often require intensive medical care, possibly in an intensive care unit (ICU). Management may involve nutritional support, organ support, and interventions to address complications.  Response 2:  Acute pancreatitis is often categorized into three severity levels—mild, moderate, and severe—based on various clinical and laboratory criteria. The severity classification helps guide treatment decisions and predict the potential complications associated with the condition. The classification systems may vary slightly between different medical organizations, but they generally include the following distinctions:  Mild Acute Pancreatitis:  Patients with mild acute pancreatitis typically exhibit the following characteristics:  Absence of organ failure.  No local or systemic complications.  Minimal or no increase in inflammatory markers (such as C-reactive protein or white blood cell count).  Generally, these patients have a straightforward and uncomplicated course, and they often recover with supportive care.  Moderate Acute Pancreatitis:  Moderate acute pancreatitis is characterized by:  Transient organ failure that resolves within 48 hours.  Local or systemic complications that may be present but are not severe.  Moderate elevations in inflammatory markers.  Some patients with moderate acute pancreatitis may require additional interventions and closer monitoring compared to those with mild pancreatitis.  Severe Acute Pancreatitis:  Severe acute pancreatitis is associated with more significant complications and organ dysfunction, including:  Persistent organ failure that lasts for more than 48 hours.  Severe local or systemic complications, such as necrosis of pancreatic or peripancreatic tissue.  Markedly elevated inflammatory markers.  Severe acute pancreatitis is often characterized by a more complicated and potentially life-threatening course, requiring intensive medical management and, in some cases, surgical intervention. | Response 1: The classification of acute pancreatitis into mild, moderate, and severe is primarily based on the Atlanta classification system, which considers the presence and persistence of organ failure and local or systemic complications.  Mild Acute Pancreatitis: This form of pancreatitis is characterized by the absence of organ failure as well as the absence of local (such as necrosis, pseudocyst, or abscess) or systemic complications.  Patients typically have minimal discomfort or pain and often recover without any lasting damage to the pancreas.  Hospitalization may be required for pain management, hydration, and nutritional support, but the length of stay is usually short.  The prognosis for mild acute pancreatitis is generally good with appropriate supportive care.  Moderately Severe Acute Pancreatitis: Moderately severe acute pancreatitis is identified by the presence of transient organ failure, which resolves within 48 hours, and/or local or systemic complications without persistent organ failure. Transient organ failure might involve temporary respiratory, cardiovascular, or renal issues that resolve with treatment.  Local complications could include fluid collections, necrosis, or the formation of pseudocysts, while systemic complications may involve exacerbations of pre-existing conditions such as diabetes. These patients may require more prolonged hospitalization and intervention, possibly including procedures to manage complications.  Severe Acute Pancreatitis: Severe acute pancreatitis is associated with persistent organ failure, defined as organ failure that lasts for more than 48 hours. It is often associated with multiple organ dysfunction syndrome (MODS), which may include persistent issues with the lungs (acute respiratory distress syndrome), kidneys (renal failure), or the cardiovascular system. There is a high risk of both local complications such as widespread pancreatic necrosis, infection, abscesses, or significant bleeding, and systemic complications that may lead to sepsis or other serious issues. These patients often require intensive care, possible mechanical ventilation, dialysis, and more invasive procedures or surgeries to address complications. Severe acute pancreatitis has a significantly higher mortality rate compared to the mild or moderately severe forms.  Response 2: The severity of acute pancreatitis can be classified into mild, moderate, and severe based on clinical findings, laboratory tests, and imaging studies. The Atlanta classification system provides the criteria for this classification:  Mild Acute Pancreatitis:  This is characterized by the absence of organ failure and local or systemic complications.  Patients may experience abdominal pain and elevations in pancreatic enzymes, but they generally have an uneventful recovery and do not suffer from long-term pancreatic dysfunction.  Hospitalization may be needed for short-term management including hydration, pain control, and nutritional support.  The prognosis is typically good, with a low risk of mortality and complications.  Moderately Severe Acute Pancreatitis:  Patients with moderately severe acute pancreatitis may develop transient organ failure (which resolves within 48 hours), local complications (such as acute peripancreatic fluid collections or pseudocysts), and/or exacerbation of pre-existing comorbid conditions.  Although there may be some complications, they are not persistent, and with treatment, patients can often recover without severe long-term consequences.  This form may require a more prolonged hospital stay and possibly procedures to manage complications.  Severe Acute Pancreatitis:  Severe acute pancreatitis is marked by persistent organ failure, which is classified when there is organ failure for more than 48 hours.  It may affect one or more organs/systems, most notably respiratory, renal, and cardiovascular systems.  Local complications can include severe pancreatic necrosis, abscesses, or significant peripancreatic fluid collections that could lead to secondary infections.  These patients are at increased risk of mortality and commonly require management in an intensive care unit, with possible interventions such as mechanical ventilation, renal replacement therapy, or surgical procedures to address complications.  The disease course is often complicated and protracted with a higher risk of both short- and long-term morbidity and mortality. | Mild acute pancreatitis： No organ failure； No local or systemic complications Moderately acute pancreatitis： Organ failure that resolves within 48 h (transient organ failure) and/or; Local or systemic complications without persistent organ failure Severe acute pancreatitis: Persistent organ failure (>48 h); Single organ failure; Multiple organ failure |
| What is the identification of two distinct phases of acute pancreatitis? | Response 1: Acute pancreatitis is often described in terms of two distinct phases: the early or acute phase and the later or recovery phase. These phases represent different stages in the course of the disease, each characterized by specific clinical and pathophysiological features.  Early (Acute) Phase: Onset of Symptoms: This phase begins with the sudden onset of abdominal pain, which is often severe and located in the upper abdomen. Other symptoms may include nausea, vomiting, and abdominal tenderness.  Inflammatory Response: The pancreas undergoes an acute inflammatory process, which can lead to the release of digestive enzymes into the pancreatic tissue, causing damage and inflammation. This phase is marked by local and systemic inflammation.  Complications: In severe cases, complications such as pancreatic necrosis, fluid collections, and organ failure may occur during the early phase.  Later (Recovery) Phase: Resolution of Acute Inflammation: With appropriate management, the acute inflammatory process begins to subside. The resolution of inflammation is associated with the healing of pancreatic tissue.  Recovery of Organ Function: If organ failure occurred during the early phase, organ function starts to improve. Patients may experience a gradual improvement in symptoms.  Management of Complications: Ongoing care focuses on managing any remaining complications, such as fluid collections or pseudocysts. Nutritional support is often an integral part of the recovery phase.  Response 2: The two distinct phases of acute pancreatitis are the early (acute) phase and the later (recovery/resolution) phase. These phases are characterized by different clinical and pathological features.  Early (Acute) Phase:  This phase is marked by the initial onset of inflammation in the pancreas.  Symptoms typically include severe abdominal pain, nausea, vomiting, and potentially fever.  Enzymes such as amylase and lipase may be elevated in blood tests.  Imaging studies, such as computed tomography (CT) scans, may reveal inflammation and swelling of the pancreas.  Complications like pancreatic necrosis, fluid collections, and systemic inflammatory response syndrome (SIRS) can occur during this phase.  Later (Recovery/Resolution) Phase:  After the initial inflammatory response, the pancreas enters a recovery phase.  In uncomplicated cases, the inflammation subsides, and the pancreas begins to heal.  Symptoms may start to improve, and laboratory tests often show a decline in enzyme levels.  In cases with complications like necrosis or pseudocysts, this phase may involve interventions such as drainage procedures or surgery.  Long-term management may be necessary to address any residual issues or prevent recurrence. | Response 1: In the clinical course of acute pancreatitis, two distinct phases are often identified. These phases are:  Early Phase: The early phase typically occurs within the first week after the onset of symptoms and is characterized by the body’s systemic inflammatory response to the pancreatic injury.  This systemic inflammatory response syndrome (SIRS) can lead to a cascade of inflammatory cytokines and can cause multi-organ dysfunction.  During this phase, patients may experience symptoms like severe abdominal pain, nausea, vomiting, fever, and elevated heart rate.  The focus of treatment during the early phase is on supportive care, which includes aggressive fluid resuscitation, management of pain, maintaining adequate nutrition, and monitoring for complications. The goal is to stabilize the patient, support organ systems, and prevent the progression to severe pancreatitis and multi-organ failure.  Late Phase:  The late phase occurs after the first week and can stretch into weeks or months following the initial symptoms.  This phase is characterized by the resolution of systemic inflammation and the development of local complications such as necrosis, abscesses, pseudocysts, or infection in or around the pancreas.  At this stage, the body's response shifts from systemic inflammation to a focus on healing and dealing with the consequences of the initial injury. Local complications may arise as a result of the damage from the early phase.  Management during the late phase shifts towards addressing these local complications. Interventional procedures may be necessary, such as drainage of fluid collections or necrosectomy in the event of infected necrosis.  Response 2: Acute pancreatitis is identified by two distinct phases:  Early Phase (Initial or Acute Phase): This phase occurs within the first week of illness and is characterized by the body's inflammatory response to pancreatic injury. The severity of acute pancreatitis in this phase is determined by the extent of the systemic inflammatory response syndrome (SIRS) and can lead to multi-organ dysfunction syndrome (MODS). The early phase involves processes such as pancreatic inflammation, edema, and possibly necrosis. Clinical features during this phase include severe abdominal pain, nausea, vomiting, fever, and elevated pancreatic enzymes in the blood.  Late Phase (Secondary or Late Onset Phase): This phase may occur more than a week after the onset of symptoms, particularly in more severe cases. It is characterized by the complications that arise from the initial inflammatory response, including infection of pancreatic or peripancreatic necrosis, persistent organ failure, and systemic complications. The late phase is often associated with the development of pancreatic pseudocysts, abscesses, and systemic complications like persistent SIRS, which can lead to a protracted hospital stay and increased mortality. | Two distinct phases of acute pancreatitis have now been identified: (i) early (within 1 week), characterized by the systemic inflammatory response syndrome (SIRS) and/or organ failure; and (ii) late ( > 1 week), characterized by local complications. |
| **Diagnosis** |  |  |  |
| What are the diagnosis criteria of acute pancreatitis? | Response 1: The diagnosis of acute pancreatitis is typically based on a combination of clinical features, laboratory tests, and imaging studies. The commonly used diagnostic criteria for acute pancreatitis include those outlined in the Revised Atlanta Classification. The diagnosis is confirmed if two of the following three criteria are present:  Characteristic Abdominal Pain:  Acute pancreatitis is often associated with severe, persistent, epigastric pain that may radiate to the back. The pain is usually sudden in onset and may be exacerbated by eating.  Elevated Serum Lipase and/or Amylase:  Laboratory tests showing elevated levels of serum lipase or amylase are a key component of the diagnosis. Lipase is more specific and remains elevated for a longer duration than amylase. A significant increase in these enzymes helps support the diagnosis of acute pancreatitis.  Imaging Findings Consistent with Acute Pancreatitis:  Radiological evidence of acute pancreatitis is often obtained through imaging studies such as abdominal ultrasound, contrast-enhanced computed tomography (CT), or magnetic resonance imaging (MRI). These studies may reveal inflammation, edema, or necrosis of the pancreas.  Response 2: The diagnosis of acute pancreatitis is typically based on a combination of clinical features, laboratory tests, and imaging studies. The commonly used criteria for diagnosing acute pancreatitis include:  Clinical Symptoms:  Severe abdominal pain, often radiating to the back.  Nausea and vomiting.  Laboratory Tests:  Elevated pancreatic enzymes: Serum amylase and lipase levels are usually elevated. Lipase is considered more specific and stays elevated for a longer duration.  Additional blood tests: Complete blood count (CBC), liver function tests, and blood glucose levels may be assessed.  Imaging Studies:  Abdominal ultrasound: This may be used to visualize the pancreas and surrounding structures. It can help identify gallstones or other causes of pancreatitis.  Contrast-enhanced computed tomography (CT) scan: This imaging technique provides detailed images of the pancreas and can help assess the severity of inflammation, identify complications, and rule out other potential causes.  Additional Criteria:  Clinical history and physical examination: Information about alcohol consumption, recent trauma, or other potential triggers can contribute to the diagnosis.  Ranson's criteria or other scoring systems: These are used to assess the severity and prognosis of acute pancreatitis based on various clinical and laboratory parameters. | Response 1: The diagnosis of acute pancreatitis typically requires the presence of at least two of the following three criteria:  Abdominal Pain: The patient usually presents with acute onset of persistent, severe epigastric pain often radiating to the back. This pain is usually intense and may be associated with nausea and vomiting.  Serum Lipase or Amylase Activity: There is an elevation in serum lipase or amylase to three times the upper limit of normal. Lipase is more specific to the pancreas and is typically preferred over amylase for diagnosis.  Imaging Findings: Abdominal imaging, like ultrasound, contrast-enhanced computed tomography (CT), magnetic resonance imaging (MRI), or endoscopic ultrasound, shows characteristic findings consistent with acute pancreatitis. These findings can include pancreatic swelling, necrosis, or changes in peripancreatic fat.  Response 2: The diagnosis of acute pancreatitis typically relies on a combination of clinical presentation, laboratory findings, and imaging studies. The most commonly used criteria for diagnosing acute pancreatitis are known as the Atlanta Criteria, which include the following:  Clinical Presentation: Acute onset of persistent, severe epigastric pain often radiating to the back. The pain is usually sudden in onset and can be associated with nausea and vomiting.  Laboratory Findings: Elevation of pancreatic enzymes in the blood, with lipase and amylase levels typically being more than three times the upper limit of normal. Lipase is more specific to the pancreas and is therefore considered more reliable for the diagnosis of acute pancreatitis.  Imaging Studies: Abdominal imaging, such as computed tomography (CT) scan, magnetic resonance imaging (MRI), or ultrasound, showing characteristic findings of acute pancreatitis. These findings can include pancreatic enlargement, inflammation, and areas of necrosis or fluid collections. | The diagnosis of acute pancreatitis requires two of the following three features: (1) abdominal pain consistent with acute pancreatitis (acute onset of a persistent, severe, epigastric pain often radiating to the back); (2) serum lipase activity (or amylase activity) at least three times greater than the upper limit of normal; and (3) characteristic findings of acute pancreatitis on contrast-enhanced computed tomography (CECT) and less commonly magnetic resonance imaging (MRI) or transabdominal ultrasonography. |
| What are the common symptoms of acute pancreatitis? | Response 1: Acute pancreatitis is characterized by the sudden onset of inflammation of the pancreas, and it often presents with a range of symptoms. The most common symptoms of acute pancreatitis include:  Abdominal Pain: Severe, persistent pain in the upper abdomen is a hallmark symptom. The pain may radiate to the back and can be aggravated by eating, especially fatty foods. Patients often describe it as a gnawing or piercing pain.  Nausea and Vomiting: Patients with acute pancreatitis may experience nausea and vomiting, often as a result of abdominal pain.  Fever and Chills: Fever and chills may occur, especially in cases where there is inflammation and infection.  Tenderness of the Abdomen: The abdomen may be tender to the touch, particularly in the upper abdominal region.  Increased Heart Rate: An elevated heart rate (tachycardia) may be observed, especially in more severe cases or when complications are present.  Hypotension: In severe cases, low blood pressure (hypotension) may occur, which could be a sign of systemic complications and organ failure.  Respiratory Symptoms: Respiratory symptoms, such as difficulty breathing, may occur in severe cases when there is involvement of the lungs or if respiratory failure develops.  Response 2: Acute pancreatitis is characterized by the sudden inflammation of the pancreas, and it presents with various symptoms. The most common symptoms of acute pancreatitis include:  Severe Abdominal Pain:  The hallmark symptom is intense, persistent abdominal pain, usually located in the upper abdomen. The pain may radiate to the back.  Nausea and Vomiting:  Individuals with acute pancreatitis often experience nausea and vomiting.  Abdominal Tenderness:  The abdomen may be tender to the touch, especially in the upper abdominal region.  Fever:  Fever is common in acute pancreatitis and may be associated with systemic inflammation.  Increased Heart Rate (Tachycardia):  Tachycardia can be a response to the inflammation and stress on the body.  Hypotension:  In severe cases, there may be a drop in blood pressure.  Jaundice:  If the inflammation affects the bile ducts, jaundice (yellowing of the skin and eyes) may occur.  Respiratory Symptoms:  In severe cases or cases with complications, respiratory symptoms may develop due to inflammation affecting the lungs. | Response 1: Common symptoms of acute pancreatitis include:  Abdominal Pain: Sudden onset of severe pain in the upper abdomen. The pain often radiates to the back and may be more intense when lying flat and better when leaning forward. It can last for several days and is typically steady and severe.  Abdominal Tenderness: The abdomen may be tender to the touch or swollen and firm (distended).  Nausea and Vomiting: These symptoms often accompany the abdominal pain.  Fever: Elevated body temperature can occur if there is inflammation or infection.  Increased Heart Rate (Tachycardia): The heart rate may increase as a response to pain or dehydration.  Jaundice: Yellowing of the skin and eyes may occur, especially if gallstones are the cause of the pancreatitis and they block the bile duct.  Changes in Blood Pressure: Blood pressure may be high or low; hypotension can occur with dehydration or if the pancreatitis is severe.  Abdominal Distension: The belly may become bloated due to ileus, where the intestines slow down or stop moving.  Changes in Bowel Movements: Due to effects on digestive processes, diarrhea or fatty stools (steatorrhea) can occur.  Response 2: Common symptoms of acute pancreatitis include:  Abdominal Pain: This is the hallmark symptom of acute pancreatitis. The pain is often severe, located in the upper abdomen, and can radiate to the back. It may become worse after eating or lying flat on the back.  Nausea and Vomiting: Many patients with acute pancreatitis experience nausea and vomiting, which can exacerbate dehydration and worsen the patient's condition.  Fever: A mild fever is not uncommon in acute pancreatitis.  Tenderness or Swelling in the Abdomen: The abdomen may be tender to the touch, and some patients may have noticeable swelling or distension.  Rapid Pulse: The heart rate may increase as the body responds to the pain and inflammation.  Jaundice: In cases where the inflammation affects the bile duct, patients may develop jaundice, leading to yellowing of the skin and eyes.  Changes in Blood Pressure: Hypotension (low blood pressure) can occur, especially in severe cases, due to fluid sequestration in the abdomen and systemic inflammatory response.  Lethargy or Weakness: Patients may feel unusually tired or weak due to the body's response to inflammation and potential nutritional deficiencies resulting from the condition. | Patients with acute pancreatitis frequently experience abdominal pain (epigastric or left upper quadrant pain), nausea, and vomiting. |
| What is the signs of acute pancreatitis systemic inflammatory response syndrome (SIRS)? | Response 1: Acute pancreatitis can lead to a systemic inflammatory response syndrome (SIRS) in more severe cases, especially when there is extensive inflammation and complications. SIRS is a clinical response to various forms of severe inflammation, and it involves a systemic activation of the immune system. The signs of SIRS associated with acute pancreatitis may include:  Fever: A body temperature above 38°C (100.4°F) or below 36°C (96.8°F) is considered one of the criteria for SIRS. In acute pancreatitis, fever is often present due to the systemic inflammatory response.  Tachycardia (Increased Heart Rate): A heart rate exceeding 90 beats per minute is a common sign of SIRS. Tachycardia may be observed in response to the inflammatory process and the body's attempt to compensate for decreased blood pressure.  Tachypnea (Increased Respiratory Rate): An elevated respiratory rate, typically more than 20 breaths per minute, can occur in response to inflammation and the need for increased oxygen delivery to tissues.  Leukocytosis (Increased White Blood Cell Count): An elevated white blood cell (WBC) count is another indicator of SIRS. In acute pancreatitis, the inflammatory response can lead to an increase in the number of circulating white blood cells.  Response 2: Acute pancreatitis can lead to the development of systemic inflammatory response syndrome (SIRS) in more severe cases. SIRS is a clinical response to systemic inflammation that can affect various organs and systems throughout the body. The signs of SIRS associated with acute pancreatitis include:  Fever or Hypothermia:  Individuals with SIRS may present with either an elevated body temperature (fever) or abnormally low body temperature (hypothermia).  Tachycardia (Increased Heart Rate):  SIRS often leads to an increased heart rate as part of the body's response to inflammation and stress.  Tachypnea (Increased Respiratory Rate):  An elevated respiratory rate may be observed, reflecting the body's attempt to compensate for the metabolic demands associated with inflammation.  Leukocytosis or Leukopenia:  There may be an abnormal increase (leukocytosis) or decrease (leukopenia) in the white blood cell count. In the context of acute pancreatitis, an elevated white blood cell count is more common.  Altered Mental Status:  In severe cases, SIRS can affect the central nervous system, leading to confusion or altered mental status. | Response 1: Acute pancreatitis can trigger a systemic inflammatory response syndrome (SIRS), which is an overwhelming inflammatory state that can affect the function of various organs. The signs of SIRS include:  Temperature: Fever (>38°C or 100.4°F) or hypothermia (<36°C or 96.8°F).  Heart Rate: Tachycardia, defined as a heart rate greater than 90 beats per minute.  Respiratory Rate: Tachypnea, with more than 20 breaths per minute, or an arterial carbon dioxide tension (PaCO2) lower than 32 mm Hg.  White Blood Cell Count: Leukocytosis (WBC count > 12,000 µL), leukopenia (WBC count < 4,000 µL), or a normal WBC count with greater than 10% immature neutrophils (band forms).  Response 2: The Systemic Inflammatory Response Syndrome (SIRS) is a widespread inflammatory response to various severe clinical insults, including acute pancreatitis. The presence of SIRS in the context of acute pancreatitis is a key indicator of the severity of the disease and can lead to severe complications, including organ failure and sepsis. The criteria for SIRS include the presence of more than one of the following clinical findings:  Temperature: Body temperature >38°C (100.4°F) or <36°C (96.8°F), indicating fever or hypothermia.  Heart Rate: Heart rate >90 beats per minute, which is a sign of tachycardia.  Respiratory Rate: Respiratory rate >20 breaths per minute or arterial carbon dioxide tension (PaCO2) <32 mm Hg, indicating tachypnea or hyperventilation. This can also be an indication of the body's attempt to compensate for metabolic acidosis.  White Blood Cell Count: Abnormal white blood cell count >12,000/µL or <4,000/µL, or the presence of more than 10% immature neutrophils (band forms). This indicates either an immune response to infection or inflammation or a suppressed immune system. | SIRS—defined by presence of two or more criteria:(1)Heart rate >90 beats/min; (2)Core temperature <36°C or >38°C; (3)White blood count <4000 or >12000/mm^3^; (4)Respirations >20/min or PCO^2^ <32 mm Hg^13^. |
| **Treatment** |  |  |  |
| What is the initial management of acute pancreatitis? | Response 1: The initial management of acute pancreatitis involves a combination of supportive care, pain control, fluid resuscitation, and identification and treatment of the underlying cause. The goals are to alleviate symptoms, prevent complications, and support the patient's recovery. Here are the key components of the initial management of acute pancreatitis:  Hospital Admission: Patients with acute pancreatitis, especially those with moderate to severe symptoms, are often admitted to the hospital for close monitoring and management.  NPO Status (Nothing by Mouth): In the early phase, patients are usually kept NPO to allow the pancreas to rest and reduce stimulation of pancreatic enzyme secretion. Enteral nutrition may be initiated if the patient cannot tolerate oral intake.  Pain Management: Pain control is a crucial aspect of management. Analgesics, such as acetaminophen or non-steroidal anti-inflammatory drugs (NSAIDs), may be used. In more severe cases, opioids may be required.  Intravenous Fluid Resuscitation: Adequate hydration is essential to prevent dehydration and maintain organ perfusion. Intravenous fluids are administered to replace fluid losses due to vomiting and prevent complications such as hypovolemic shock.  Electrolyte Monitoring and Correction: Close monitoring of electrolyte levels, particularly calcium and magnesium, is important. Imbalances can occur due to the release of pancreatic enzymes.  Identifying and Treating Underlying Causes: The underlying cause of acute pancreatitis should be identified and addressed. Common causes include gallstones, alcohol consumption, medications, and other factors. Specific interventions may include cholecystectomy for gallstone pancreatitis or alcohol cessation counseling.  Monitoring for Complications: Regular monitoring for complications, such as respiratory distress, organ failure, or signs of infection, is crucial. In severe cases, patients may require admission to an intensive care unit (ICU).  Nutritional Support: Nutritional support is important, especially in severe cases. Enteral nutrition is preferred over parenteral nutrition, and it may be initiated when the patient is stable.  Avoiding Triggers: Patients are advised to avoid alcohol and fatty foods during the acute phase to reduce stress on the pancreas.  Response 2: The initial management of acute pancreatitis involves a combination of supportive care, pain management, and addressing potential underlying causes. The goals are to alleviate symptoms, prevent complications, and support the healing process. Here are key aspects of the initial management:  Hospitalization:  Most cases of acute pancreatitis require hospitalization, especially if the symptoms are severe or if complications are present.  NPO Status (Nothing by Mouth):  Initially, patients are often kept NPO to allow the pancreas to rest and reduce stimulation of pancreatic enzyme secretion. Nutritional support may be introduced gradually as tolerated.  Intravenous (IV) Fluids:  Adequate hydration is crucial to prevent dehydration and support the cardiovascular system. Intravenous fluids are administered to maintain fluid and electrolyte balance.  Pain Management:  Pain control is essential. Analgesics, such as acetaminophen or nonsteroidal anti-inflammatory drugs (NSAIDs), may be used. In more severe cases, opioids may be necessary.  Addressing Underlying Causes:  Identification and treatment of the underlying cause of pancreatitis are essential. This may include addressing gallstones, alcohol withdrawal, or discontinuing medications that could be contributing.  Monitoring and Laboratory Tests:  Regular monitoring of vital signs, fluid balance, and laboratory tests (including pancreatic enzymes, blood counts, and electrolytes) helps assess the patient's condition and guide treatment adjustments.  Nutritional Support:  Nutritional support is gradually introduced as the patient's condition improves. Enteral nutrition (feeding through a tube into the stomach or small intestine) is preferred over total parenteral nutrition (IV feeding) in most cases.  Consideration of Complications:  Complications such as pancreatic necrosis, pseudocysts, or infected fluid collections may require specific interventions, including drainage procedures or surgery.  Avoiding Alcohol and Smoking:  Patients with alcoholic pancreatitis are advised to abstain from alcohol, and smoking cessation is recommended. | Response 1: Initial management of acute pancreatitis generally involves supportive care to stabilize the patient, relieve symptoms, and prevent complications. The basic principles of the initial treatment for acute pancreatitis are:  Fasting (NPO - Nothing by mouth): Patients are typically kept NPO to rest the pancreas and reduce the stimulation of pancreatic enzyme secretions that can exacerbate inflammation.  Aggressive Hydration: Intravenous (IV) fluid resuscitation is critical, especially within the first 24-48 hours, unless contraindicated by renal or cardiac conditions.  Pain Management: Pain control is achieved with analgesics, with opioids often used for severe pain. Adequate pain control is essential but should be balanced against the risk of respiratory depression.  Nutritional Support: Enteral nutrition (tube feeding) should be initiated as early as possible, typically within 48-72 hours, if the patient is unable to eat. Parenteral nutrition (IV nutrition) is considered if enteral nutrition is not tolerated.  Monitoring and Managing Complications: Continuous monitoring for signs of complications such as organ failure is essential. Treatment of complications is guided by the specific issue, such as administration of antibiotics for infected necrotizing pancreatitis or interventions for acute fluid collections.  Managing Underlying Causes: If the pancreatitis is due to gallstones (gallstone pancreatitis), surgical or endoscopic intervention to remove the gallstones may be indicated, usually after inflammation has subsided. If alcohol is the cause, abstinence and counseling are recommended.  Laboratory and Imaging Studies: Monitoring of relevant laboratory values (such as amylase, lipase, complete blood count, liver enzymes, kidney function, and electrolytes) and imaging studies to monitor the progression of the disease.  Oxygen Support: Supplemental oxygen is provided to patients with hypoxia.  Response 2: The initial management of acute pancreatitis focuses on supportive care to alleviate symptoms, prevent complications, and address the underlying cause of the inflammation. The mainstays of initial management include:  Fluid Resuscitation: Aggressive intravenous hydration is crucial, especially in the first 24-48 hours, to counteract the effects of fluid loss and prevent hypovolemia. Lactated Ringer's solution is often preferred due to its potential benefits in the inflammatory response.  Pain Management: Pain in acute pancreatitis can be severe. Analgesics, typically starting with non-opioid analgesics and progressing to opioids if necessary, are used to control pain. Patient-controlled analgesia (PCA) may be beneficial for some patients.  Nutritional Support: Early enteral nutrition (feeding through the gastrointestinal tract) is recommended within 24-48 hours if the patient can tolerate it, as it has been shown to reduce complications. If enteral nutrition is not possible, parenteral nutrition (intravenous feeding) may be considered, although it's generally less preferred due to higher complication rates.  NPO Status (Nothing by Mouth): Initially, patients are often kept NPO (nothing by mouth) to rest the pancreas and reduce pancreatic secretions. The duration of this management approach depends on the patient's symptoms and clinical improvement.  Monitoring: Close monitoring of vital signs, fluid balance, and laboratory values (including electrolytes, renal function, and markers of inflammation) is essential to detect and manage complications early.  Treatment of Underlying Causes: Addressing the underlying causes of acute pancreatitis, such as gallstones or alcohol use, is critical. This may involve surgical or endoscopic interventions, such as cholecystectomy for gallstones or endoscopic retrograde cholangiopancreatography (ERCP) for bile duct obstructions.  Avoidance of Alcohol and Smoking: Patients are advised to abstain from alcohol and smoking to reduce the risk of recurrent episodes and complications.  Antibiotics: The use of antibiotics is controversial and generally not recommended unless there is evidence of infection, such as infected pancreatic necrosis.  ICU Admission: Patients with severe acute pancreatitis, especially those exhibiting signs of systemic inflammatory response syndrome (SIRS) or organ failure, may require admission to an intensive care unit (ICU) for more intensive monitoring and management. | Fluid therapy to prevent hypovolemia and organ hypoperfusion is a long-established cornerstone of the initial management of AP. |
| When should patients with acute pancreatitis combined with acute cholangitis receive Endoscopic Retrograde Cholangiopancreatography (ERCP)? | Response 1: Endoscopic Retrograde Cholangiopancreatography (ERCP) is a procedure used to diagnose and treat conditions of the bile ducts and pancreas. In the context of acute pancreatitis combined with acute cholangitis, ERCP is often considered an important intervention. The decision to perform ERCP in these cases is guided by certain clinical indications and timing considerations. The primary goal is to relieve biliary obstruction and prevent further complications. Here are general guidelines for the timing of ERCP in patients with acute pancreatitis and acute cholangitis:  Urgent or Emergency ERCP: Within 24-72 hours: In cases of acute cholangitis with evidence of biliary obstruction (e.g., jaundice, elevated liver enzymes) and concomitant acute pancreatitis, urgent or emergency ERCP is often recommended. Early intervention helps to relieve the obstruction, reduce the risk of complications, and improve outcomes.  High-Risk Features: As soon as possible: If there are high-risk features such as sepsis, hemodynamic instability, or evidence of organ failure, ERCP should be performed as soon as possible. These patients may require intensive care unit (ICU) admission for close monitoring.  Timing Considerations: Early intervention: ERCP is generally considered in the early phase of acute pancreatitis, particularly if there is evidence of biliary obstruction contributing to the disease. Early intervention can help prevent worsening of pancreatitis and reduce the risk of complications.  Imaging Confirmation: Based on imaging findings: ERCP is typically guided by imaging studies such as abdominal ultrasound, computed tomography (CT), or magnetic resonance cholangiopancreatography (MRCP). Imaging can help confirm the presence of biliary obstruction and guide the decision to perform ERCP.  Consideration of Endoscopic Sphincterotomy (ES): For stone extraction: If common bile duct stones are identified as the cause of biliary obstruction, endoscopic sphincterotomy (ES) during ERCP may be performed to facilitate stone extraction.  Response 2: Patients with acute pancreatitis combined with acute cholangitis often require prompt intervention, and Endoscopic Retrograde Cholangiopancreatography (ERCP) is a key therapeutic procedure in such cases. The decision to perform ERCP is based on clinical assessment, laboratory findings, and imaging studies. The general recommendation is to perform ERCP as soon as possible, typically within 24 to 72 hours, in patients with acute pancreatitis and acute cholangitis.  Indications for urgent or emergent ERCP in the setting of acute pancreatitis and acute cholangitis include:  Evidence of Biliary Obstruction: When there is clear evidence of biliary obstruction contributing to acute pancreatitis and acute cholangitis, prompt relief of the obstruction is essential. This is often confirmed by imaging studies, such as abdominal ultrasound, magnetic resonance cholangiopancreatography (MRCP), or other modalities.  Clinical Signs of Acute Cholangitis: The presence of clinical signs of acute cholangitis, such as Charcot's triad (fever, right upper quadrant pain, and jaundice) or Reynolds' pentad (Charcot's triad plus hypotension and altered mental status), indicates a severe and potentially life-threatening condition requiring urgent intervention.  High-Risk Features: Patients with high-risk features, including sepsis, hemodynamic instability, or evidence of organ failure, require early and aggressive management. ERCP plays a crucial role in addressing the underlying biliary obstruction in such cases.  Imaging Confirmation: Imaging studies, such as MRCP or contrast-enhanced CT scan, can help confirm the presence and location of biliary obstruction and guide the decision to proceed with ERCP.  Prevention of Complications: Early intervention with ERCP is aimed at preventing complications associated with acute cholangitis, such as progression to septic shock, multiple organ failure, and other serious consequences.  Determination of Underlying Cause: ERCP not only provides therapeutic relief of biliary obstruction but also allows for the identification of the underlying cause, such as common bile duct stones, which can then be addressed during the procedure. | Response 1: Patients with acute pancreatitis combined with acute cholangitis should typically receive Endoscopic Retrograde Cholangiopancreatography (ERCP) urgently, often within 24 to 48 hours of presentation, especially if there is evidence of ongoing biliary obstruction. Cholangitis is an infection of the bile duct, which can be life-threatening if not treated promptly. Indications for urgent ERCP in patients with acute pancreatitis and concomitant acute cholangitis include:  Clinical Signs of Cholangitis, such as Charcot's triad (jaundice, fever or chills, and right upper quadrant pain) or Reynold's pentad, which adds hypotension and altered mental status as signs of cholangitis with sepsis.  Evidence of Biliary Obstruction on imaging, such as dilated bile ducts or the presence of stones in the common bile duct.  Abnormal Liver Function Tests that suggest biliary obstruction, such as increased bilirubin, alkaline phosphatase (ALP), gamma-glutamyl transferase (GGT), and transaminases.  Response 2: For patients with acute pancreatitis combined with acute cholangitis, Endoscopic Retrograde Cholangiopancreatography (ERCP) is considered an urgent intervention. The timing for ERCP in this scenario is usually within 24 hours of diagnosis, particularly in cases exhibiting signs of cholangitis severity such as systemic inflammation, cholestasis (elevated bilirubin levels and liver enzymes), and evidence of bile duct obstruction on imaging.  The indications for urgent ERCP in the context of acute pancreatitis with cholangitis include:  Clinical signs of cholangitis: These can include Charcot's triad of jaundice, fever (or chills), and right upper quadrant pain. Reynold's pentad, which adds hypotension and altered mental status to Charcot's triad, indicates severe cholangitis.  Evidence of biliary obstruction: Imaging studies (e.g., ultrasound, CT scan, or MRI) showing dilated bile ducts or the presence of stones in the common bile duct suggest obstruction.  Laboratory evidence of cholestasis: Elevated levels of bilirubin and liver enzymes such as alkaline phosphatase and gamma-glutamyl transferase (GGT) can indicate cholestasis and biliary obstruction. | Patients with acute pancreatitis and concurrent acute cholangitis should undergo ERCP within 24 hours of admission. |
| What should be done for an acute pancreatitis extrapancreatic infection, such as cholangitis, catheter-acquired infections, bacteremia, urinary tract infections, and pneumonia? | Response 1: The presence of extrapancreatic infections in the context of acute pancreatitis can significantly impact the course of the disease and increase the risk of complications. Management of extrapancreatic infections associated with acute pancreatitis involves a combination of supportive care, antimicrobial therapy, and specific interventions based on the nature of the infection. Here are general considerations for managing various extrapancreatic infections in the setting of acute pancreatitis:  Cholangitis (Biliary Infection): ERCP and Biliary Drainage: If cholangitis is suspected or confirmed, early Endoscopic Retrograde Cholangiopancreatography (ERCP) with biliary drainage is often warranted. This helps to relieve biliary obstruction and improve the clearance of infected material.  Catheter-Acquired Infections: Removal or Replacement of Catheters: If the patient has catheter-acquired infections, the source of infection (e.g., central venous catheter or urinary catheter) should be addressed. Consideration may be given to removing or replacing the catheter, and appropriate antimicrobial therapy should be initiated.  Bacteremia: Antimicrobial Therapy: Treatment of bacteremia associated with acute pancreatitis involves the administration of appropriate antimicrobial therapy. The selection of antibiotics should be based on blood culture results and local antibiograms.  Urinary Tract Infections (UTIs): Antimicrobial Therapy: UTIs should be treated with appropriate antimicrobial agents based on urine culture results. Adequate hydration is important to flush out bacteria from the urinary tract.  Pneumonia: Respiratory Support and Antibiotics: In cases of pneumonia, respiratory support may be necessary, and appropriate antibiotics should be initiated based on the suspected or confirmed pathogens.  Supportive Care: Fluid Resuscitation: Adequate intravenous fluid resuscitation is crucial to maintain organ perfusion and support the immune response in the presence of infections.  Monitoring and Close Observation: Clinical Monitoring: Patients with extrapancreatic infections should be closely monitored for signs of clinical improvement or deterioration. Continuous assessment of vital signs, laboratory parameters, and imaging findings is essential.  Collaboration with Infectious Disease Specialists: Consultation: In complex cases or cases with resistant organisms, consultation with infectious disease specialists can be beneficial to optimize antimicrobial therapy.  Response 2: I am not a doctor, but I can provide some general information. It's crucial to consult with a healthcare professional for advice tailored to your specific situation. In cases of acute pancreatitis with extrapancreatic infections, such as cholangitis, catheter-acquired infections, bacteremia, urinary tract infections, and pneumonia, the management typically involves addressing the underlying cause and administering appropriate treatments. Here are some general considerations:  Identification of the source of infection: Thorough diagnostic tests, such as blood cultures, imaging studies, and other relevant tests, may be conducted to identify the specific source of infection.  Antibiotic therapy: Based on the identified pathogens and their sensitivity, appropriate antibiotics may be prescribed. The choice of antibiotics should be guided by the type of infection and local resistance patterns.  Supportive care: Patients with acute pancreatitis often require supportive care, including pain management, fluid resuscitation, and nutritional support. This helps to maintain overall health and aid in the recovery process.  Interventional procedures: If an infection is related to biliary obstruction or gallstones, interventions such as endoscopic retrograde cholangiopancreatography (ERCP) or surgery may be considered to address the underlying issue.  Monitoring and follow-up: Close monitoring of the patient's clinical status, laboratory parameters, and imaging studies is essential to assess the response to treatment. Adjustments to the treatment plan may be necessary based on the patient's progress.  Prevention of complications: Efforts should be made to prevent complications associated with infections, such as sepsis or organ failure. Early detection and management of complications are crucial for a better prognosis.  Multidisciplinary approach: Collaboration among different medical specialists, including gastroenterologists, infectious disease specialists, and surgeons, may be necessary for comprehensive care. | Response 1: If a patient with acute pancreatitis develops extrapancreatic infections such as cholangitis, catheter-acquired infections, bacteremia, urinary tract infections, or pneumonia, the following steps should be taken:  Prompt Identification: Monitor for clinical signs of infection, including fever, elevated white blood cell count, new onset of organ dysfunction, or localized symptoms like right upper quadrant pain in cholangitis.  Perform appropriate imaging studies and laboratory tests to identify potential sources of infection.  Sampling and Cultures: Obtain samples for culture before starting antibiotic therapy whenever possible, including blood cultures, urine cultures, sputum cultures, or other relevant specimens depending on the suspected source of infection.  Antibiotic Therapy: Initiate empiric broad-spectrum antibiotics promptly after obtaining cultures, as delays in treatment may worsen outcomes.  The choice of antibiotics should cover the likely pathogens associated with the specific infectious process. For instance, in cholangitis, antibiotics should target enteric bacteria, while in pneumonia, coverage should include typical respiratory pathogens.  Antibiotics may be adjusted based on culture results and antibiotic sensitivity patterns once available.  Source Control: Any identified source of infection, such as gallstones causing cholangitis or an infected catheter, should be addressed promptly. This may require procedures such as ERCP for cholangitis or catheter replacement for catheter-related infections.  Drainage of infected fluid collections through percutaneous or endoscopic approaches may be necessary if there is evidence of infected pancreatic necrosis or abscesses.  Supportive Care: Provide supportive care for organ systems affected by the infection—for example, renal replacement therapy for renal failure or mechanical ventilation for respiratory failure.  Monitor for signs of sepsis and initiate sepsis management protocols if necessary.  Review Antibiotic Use: After initial empiric treatment, review the antibiotic regimen based on culture results, clinical response, and expert recommendations.  De-escalate antibiotics to narrow-spectrum agents when possible to reduce the development of antibiotic resistance.  Prevention of Future Infections: Utilize aseptic techniques for catheter insertion and maintenance.  Practice good hand hygiene and follow infection control protocols to prevent nosocomial infections.  Review and remove invasive devices as soon as they are no longer required.  Response 2: Management of extrapancreatic infections in the context of acute pancreatitis, including cholangitis, catheter-acquired infections, bacteremia, urinary tract infections (UTIs), and pneumonia, involves several key strategies:  Prompt Identification and Diagnosis:  Clinical Assessment: Regular monitoring of patients for signs and symptoms of infection, such as fever, altered mental status, hypotension, dysuria, or respiratory distress.  Laboratory Tests: Complete blood count (CBC) with differential, blood cultures, urine analysis, and cultures, as well as sputum cultures for patients with respiratory symptoms.  Imaging Studies: Depending on the suspected site of infection, imaging studies such as chest X-ray or CT scans of the chest/abdomen can help identify pneumonia, cholangitis, or other sources of infection.  Antibiotic Therapy:  Empirical Antibiotics: Initiate broad-spectrum antibiotics empirically based on the suspected source of infection and local antibiotic resistance patterns, adjusting as necessary when culture and sensitivity results are available.  Targeted Therapy: Once specific pathogens are identified, antibiotics should be tailored to target those organisms effectively.  Source Control:  ERCP for Cholangitis: For cholangitis, ERCP with sphincterotomy and stone extraction or stenting can be performed to relieve biliary obstruction.  Drainage of Collections: Abscesses or infected fluid collections may require percutaneous or surgical drainage.  Catheter Management: For catheter-acquired infections, removal or replacement of the catheter, along with appropriate antibiotic treatment, is crucial.  Supportive Care:  Fluid Resuscitation: Ensure adequate hydration and electrolyte balance.  Pain Management: Address pain while minimizing the use of medications that may affect organ function.  Nutritional Support: Maintain nutritional status through enteral nutrition if possible, or parenteral nutrition if necessary.  Prevention of Further Infections:  Strict Asepsis: Adhere to strict aseptic techniques for the placement and care of invasive devices.  Prophylaxis: In some cases, prophylactic antibiotics may be considered to prevent infections, although this approach is subject to ongoing debate and should be tailored to individual patient risk factors.  Monitoring and Follow-up:  Clinical Monitoring: Regular assessment of the patient's clinical status to detect any signs of worsening infection or the development of new infections.  Laboratory and Imaging Follow-up: Repeat cultures, blood tests, and imaging studies as necessary to monitor the response to treatment. | Antibiotics should be given for an extrapancreatic infection, such as cholangitis, catheter-acquired infections, bacteremia, urinary tract infections, and pneumonia. |
| Should prophylactic antibiotics be routinely used in patients with severe acute pancreatitis? | Response 1: The use of prophylactic antibiotics in patients with severe acute pancreatitis remains a topic of debate in the medical literature. Prophylactic antibiotics are not routinely recommended for all patients with severe acute pancreatitis, and their use should be considered carefully based on the individual patient's risk factors and clinical presentation.  The decision to administer prophylactic antibiotics in severe acute pancreatitis is influenced by several factors, including the risk of infection, the presence of infected necrosis, and the overall severity of the disease. Key considerations include:  Infected Necrosis Risk: Prophylactic antibiotics are more commonly considered in cases where there is a high risk of infected pancreatic or peripancreatic necrosis. Infected necrosis is associated with increased morbidity and mortality, and antibiotics may be used to reduce the risk of infection or to manage documented infection.  Necrotizing Pancreatitis: In patients with necrotizing pancreatitis, especially if there is evidence of systemic inflammatory response syndrome (SIRS), organ failure, or other signs of severe disease, prophylactic antibiotics may be considered to reduce the risk of secondary infection.  Timing of Antibiotic Administration: The optimal timing for the administration of prophylactic antibiotics is an area of ongoing research. Some studies suggest that early initiation of antibiotics within the first 72 hours of symptom onset may be more beneficial, while others argue for a more selective approach based on clinical and imaging findings.  Selective Use: The use of prophylactic antibiotics is often selective and based on individual patient risk factors. Factors such as the severity of pancreatitis, the presence of necrosis, and signs of infection guide the decision-making process.  Guidelines and Recommendations: Guidelines from professional medical organizations, such as the American Gastroenterological Association (AGA) and the International Association of Pancreatology (IAP), may provide recommendations on the use of prophylactic antibiotics in severe acute pancreatitis. It is essential to consider the most recent and relevant guidelines.  Response 2: The use of prophylactic antibiotics in patients with severe acute pancreatitis remains a topic of debate in the medical community, and recommendations may vary. Prophylactic antibiotics are antibiotics given to prevent infection rather than to treat an established infection.  The decision to use prophylactic antibiotics in severe acute pancreatitis depends on various factors, including the patient's overall health, the presence of infected necrosis or other complications, and local guidelines. Here are some considerations:  Infected necrosis:  If there is evidence of infected pancreatic or peripancreatic necrosis, antibiotic therapy is generally recommended. In this case, antibiotics are used as part of the treatment strategy to manage the infection.  Risk of infection:  Patients with severe acute pancreatitis are at an increased risk of developing infections, especially if there is necrotic tissue. Prophylactic antibiotics may be considered in certain cases to reduce the risk of infection.  Local guidelines:  Medical practices can vary, and local guidelines or institutional protocols may influence whether prophylactic antibiotics are routinely used in cases of severe acute pancreatitis.  Individual patient factors:  The decision to use prophylactic antibiotics should take into account the patient's overall health, comorbidities, and the presence of other risk factors for infection.  Balancing risks and benefits:  There is ongoing research to determine the optimal use of prophylactic antibiotics in severe acute pancreatitis. The potential benefits of preventing infection need to be weighed against the risks of antibiotic resistance and other side effects associated with prolonged antibiotic use. | Response 1: The use of prophylactic antibiotics in severe acute pancreatitis has been a topic of considerable debate. The current consensus and clinical guidelines generally advise against the routine use of prophylactic antibiotics for all patients with severe acute pancreatitis. This recommendation is based on several factors:  Lack of Evidence for Efficacy: Clinical studies and meta-analyses have not consistently shown that prophylactic antibiotics reduce the risk of pancreatic infection or overall mortality in severe acute pancreatitis.  Antibiotic Resistance: The unnecessary use of antibiotics can contribute to the development of antibiotic-resistant bacteria, which is a significant public health concern.  Alteration of Gut Microflora: Antibiotics can disrupt the normal gut flora, potentially leading to other complications such as Clostridioides difficile (formerly known as Clostridium difficile) infection.  Potential Side Effects: Long-term antibiotic use may increase the risk of side effects and adverse drug reactions.  Response 2:  The use of prophylactic antibiotics in patients with severe acute pancreatitis has been a topic of considerable debate and research. The primary concern has been the prevention of infected pancreatic necrosis, which is a serious and potentially fatal complication. However, the current consensus and guidelines, based on evidence from clinical trials and meta-analyses, generally recommend against the routine use of prophylactic antibiotics in severe acute pancreatitis for several reasons:  Lack of Evidence for Benefit: Several studies and systematic reviews have found that the routine use of prophylactic antibiotics does not significantly reduce the risk of infected pancreatic necrosis or mortality in patients with severe acute pancreatitis.  Antibiotic Resistance: The indiscriminate use of antibiotics can contribute to the development of antibiotic-resistant bacteria, making infections more difficult to treat.  Fungal Infections: There is evidence suggesting that the use of prophylactic antibiotics may increase the risk of fungal infections in patients with acute pancreatitis.  Selection of Patients: Some experts suggest that rather than broad application, a more targeted approach might be beneficial. Prophylactic antibiotics may be considered in certain high-risk patients, such as those with extensive pancreatic necrosis seen on imaging, although this approach remains controversial and is not widely recommended without clear evidence of infection. | Routine use of prophylactic antibiotics in patients with severe acute pancreatitis is not recommended. |
| Should patients with sterile necrosis use antibiotics to prevent the development of infected necrosis? | Response 1: The use of antibiotics to prevent the development of infected necrosis in patients with sterile necrosis (necrosis without evidence of infection) is a topic of debate in the medical literature, and there is no clear consensus on the optimal approach. As of my knowledge cutoff in January 2022, the most recent guidelines and studies suggested a selective and cautious approach to antibiotic prophylaxis in patients with sterile necrosis. Recommendations may evolve, so it's essential to consider the latest guidelines and evidence.  Key considerations regarding the use of antibiotics in patients with sterile necrosis in the context of acute pancreatitis include:  Risk of Infection: Sterile necrosis has a lower risk of infection compared to infected necrosis. In the absence of signs and symptoms of infection, some experts argue against routine antibiotic prophylaxis to avoid unnecessary exposure to antibiotics.  Selective Use: The decision to use antibiotics may be more selective, with consideration given to patients who develop signs and symptoms of infection during the course of their illness. Close monitoring for signs of infection is crucial.  Clinical and Radiological Assessment: Regular clinical and radiological assessments are essential to monitor the patient's condition. If signs of infection (such as fever, leukocytosis, or deteriorating clinical status) or radiological evidence of infection (gas within necrotic areas) emerge, antibiotic therapy may be initiated.  Early Intervention if Infection Develops: If infection develops, early initiation of antibiotic therapy is crucial to manage the infection and reduce the risk of complications. Imaging studies, such as contrast-enhanced CT scans, may help in identifying infection.  Guidelines and Expert Consensus: Some guidelines and expert consensus statements may provide recommendations on the use of antibiotics in the context of acute pancreatitis with necrosis. It is important to consider the most recent and relevant guidelines.  Individualized Approach: The decision to use antibiotics in patients with sterile necrosis should be individualized, taking into account the patient's overall clinical condition, comorbidities, and the local prevalence of resistant organisms.  Response 2: The use of antibiotics in patients with sterile necrosis in acute pancreatitis is a subject of debate, and recommendations may vary among healthcare professionals and medical guidelines. Sterile necrosis refers to necrotic tissue without evidence of infection.  As of my knowledge cutoff in January 2022, the approach to antibiotic use in patients with sterile necrosis is not universally agreed upon. Some considerations in this context include:  Watchful waiting:  In some cases of sterile necrosis, a conservative approach without the immediate use of antibiotics may be adopted. The idea is to monitor the patient closely for signs of infection and initiate antibiotic therapy if infection develops.  Selective use of antibiotics:  Antibiotics may be selectively used in patients with sterile necrosis if there are specific risk factors for infection or if there is a concern that infection might develop. The decision to use antibiotics is often individualized based on the patient's clinical condition, imaging findings, and other factors.  Avoidance of unnecessary antibiotics:  Given concerns about antibiotic resistance and potential adverse effects, there is an emphasis on avoiding unnecessary antibiotic use. Using antibiotics only when there is a clear indication, such as the development of infection, helps prevent unnecessary exposure.  Local guidelines and practices:  Practices may vary among healthcare institutions and regions. Local guidelines and protocols may influence whether antibiotics are routinely prescribed in cases of sterile necrosis. | Response 1: Patients with sterile necrosis in the context of acute pancreatitis generally do not require prophylactic antibiotics to prevent the development of infected necrosis. Current clinical practice guidelines suggest the following:  Observation and Monitoring:  Patients with sterile pancreatic necrosis should be closely monitored for clinical signs of infection and systemic inflammatory response.  Selective Antibiotic Use:  Antibiotics are indicated if there is evidence of infection based on clinical signs, such as fever or leukocytosis, and imaging findings, such as gas in the pancreatic or peripancreatic tissue.  Minimal Intervention:  Interventional procedures or surgery should be avoided unless there is a clear indication, such as confirmed infection or symptoms that are not alleviated by conservative management. The reason for this conservative approach is to allow the area of necrosis to become "walled-off," which can make any future necessary interventions safer and more effective.  Supportive Care:  Supportive care is critical and includes fluid resuscitation, nutritional support, and pain management to allow the pancreas to heal on its own if possible.  Response 2: The use of antibiotics specifically to prevent the development of infected necrosis in patients with sterile pancreatic necrosis is generally not recommended, based on current evidence and guidelines. The rationale behind this approach includes:  Lack of Proven Benefit: Research has not definitively shown that prophylactic antibiotics in patients with sterile necrosis effectively prevent the development of infected necrosis. Clinical trials and meta-analyses have failed to demonstrate a clear benefit in reducing the incidence of infected necrosis or improving overall outcomes with the prophylactic use of antibiotics.  Risk of Antibiotic Resistance: The unnecessary use of antibiotics can contribute to the development of antibiotic-resistant bacteria. This is a significant concern in hospital settings, where multidrug-resistant organisms can complicate the treatment of infections.  Potential for Fungal Infections: There is evidence that the use of prophylactic antibiotics may increase the risk of secondary fungal infections in patients with acute pancreatitis, which can be challenging to treat.  Recommendations from Guidelines: Major guidelines, such as those from the American Gastroenterological Association (AGA) and other international gastroenterology societies, advise against the routine use of prophylactic antibiotics in patients with acute pancreatitis, including those with sterile necrosis. | The use of antibiotics in patients with sterile necrosis to prevent the development of infected necrosis is not recommended. |
| Should patients with mild acute pancreatitis found to have gallstones in the gallbladder receive cholecystectomy before discharge？ | Response 1: The decision to perform cholecystectomy in patients with mild acute pancreatitis and concurrent gallstones is typically individualized based on various factors. The general approach to cholecystectomy in the context of mild acute pancreatitis and gallstones may involve the following considerations:  Timing of Cholecystectomy: In many cases, cholecystectomy is not performed during the acute episode of mild pancreatitis but is planned for a later date, often during the same hospital admission or in the weeks following discharge.  Resolution of Acute Pancreatitis: The acute episode of pancreatitis needs to resolve before elective cholecystectomy is considered. The resolution of symptoms, normalization of pancreatic enzymes, and improvement of the patient's overall condition are important factors.  Individualized Assessment:  The decision on the timing of cholecystectomy is based on an individualized assessment of the patient's health, comorbidities, and the severity of the pancreatitis episode.  Factors Influencing Timing: Factors influencing the timing of cholecystectomy may include the patient's overall stability, the presence of complications, the severity of gallstone disease, and the potential for recurrent episodes of pancreatitis.  Guidelines and Recommendations: Some guidelines, such as those from the American College of Gastroenterology (ACG), suggest that early cholecystectomy (within 2 to 4 weeks after resolution of pancreatitis) may be considered in patients with mild gallstone pancreatitis.  Preventing Recurrence: Cholecystectomy is recommended to prevent recurrence of gallstone-related pancreatitis. However, the urgency of the procedure depends on the specific clinical scenario.  Patient Education and Informed Consent: Patients should be educated about the benefits and risks of cholecystectomy, and informed consent should be obtained before the procedure.  Response 2: The decision to perform cholecystectomy (surgical removal of the gallbladder) in patients with mild acute pancreatitis and gallstones is generally based on several factors, including the patient's overall health, the severity of the pancreatitis, and the likelihood of recurrent attacks. While there is no one-size-fits-all answer, here are some considerations:  Timing of Cholecystectomy:  In many cases, cholecystectomy is not performed during the acute phase of pancreatitis. Instead, it may be delayed until the patient has recovered from the acute episode, inflammation has subsided, and the patient is stable for surgery.  Individual Assessment:  Each case should be evaluated individually. Factors such as the patient's age, comorbidities, the presence of complications, and the cause of pancreatitis play a role in the decision-making process.  Recurrent Pancreatitis Risk:  The presence of gallstones is a common cause of pancreatitis. If gallstones are identified as the cause, and the patient has experienced an episode of mild acute pancreatitis, there is a risk of recurrent pancreatitis until the gallbladder is removed.  Preventing Recurrent Attacks:  Cholecystectomy is often recommended to prevent recurrent episodes of pancreatitis caused by gallstones. This is particularly relevant if the patient is at a higher risk of complications or if there is evidence that gallstones contributed to the pancreatitis episode.  Patient Stability:  The decision to perform cholecystectomy is influenced by the patient's overall stability. If the patient is medically stable, the surgery may be performed before discharge. However, if the patient is not stable or there are other complications, a delayed approach may be preferred.  Conservative Management:  In some cases, a more conservative approach may be taken, and the decision to proceed with cholecystectomy may be made on an outpatient basis after the patient has recovered from the acute episode. | Response 1: Yes, patients with mild acute pancreatitis who are found to have gallstones in the gallbladder (gallstone pancreatitis) should typically undergo cholecystectomy before discharge from the hospital. The rationale for this recommendation is to prevent recurrent episodes of gallstone pancreatitis, which can be severe and life-threatening.  The timing of the cholecystectomy can vary based on several factors, including the patient's clinical stability and local practice patterns. However, current guidelines often recommend that cholecystectomy be performed during the same hospital admission for pancreatitis, ideally after the acute inflammation has resolved and the patient is clinically stable, which is often within 48 to 72 hours of admission.  For patients who are not good surgical candidates or those who decline surgery, alternative treatments like endoscopic biliary sphincterotomy may be considered to reduce the risk of recurrence, although this approach is generally less definitive than cholecystectomy in terms of preventing future episodes.  It's important to individually assess each patient's situation and risks. The decision to perform cholecystectomy should involve a thorough discussion between the patient, the gastroenterologist and the surgeon, taking into account the patient's overall health, the severity of pancreatitis, and any comorbid conditions.  Response 2: For patients with mild acute pancreatitis caused by gallstones (gallstone pancreatitis), current guidelines recommend cholecystectomy (surgical removal of the gallbladder) during the same hospital admission, ideally within 48 hours of admission if the patient's condition is stable and there are no contraindications to surgery. This recommendation is based on evidence suggesting that early cholecystectomy significantly reduces the risk of recurrent gallstone-related events, such as pancreatitis, cholecystitis, or biliary colic, and potentially avoids the need for readmission for surgery.  Key points supporting early cholecystectomy in mild gallstone pancreatitis include:  Prevention of Recurrence: Early cholecystectomy has been shown to prevent recurrence of gallstone pancreatitis by addressing the underlying cause—gallstones.  Safety and Efficacy: In patients with mild acute pancreatitis, cholecystectomy is considered safe and effective when performed by experienced surgeons.  Reduced Hospital Stay and Costs: Early intervention may also reduce the overall length of hospital stay and healthcare costs by preventing recurrent episodes and complications that require readmission or additional treatments.  Timing: The optimal timing for surgery after the resolution of acute inflammatory symptoms is still debated, but the consensus leans towards early surgery during the same admission for mild cases. Delaying surgery, particularly beyond the initial hospitalization, is associated with a higher risk of gallstone-related complications. | In patients with mild acute pancreatitis, found to have gallstones in the gallbladder, a cholecystectomy should be performed before discharge to prevent a recurrence of acute pancreatitis. |
| Does the presence of asymptomatic pseudocysts and pancreatic and/or extrapancreatic necrosis require intervention？ | Response 1: The management of asymptomatic pseudocysts and pancreatic and/or extrapancreatic necrosis in acute pancreatitis depends on various factors, including the clinical presentation, size and characteristics of the pseudocysts or necrosis, and the overall condition of the patient. Not all cases require immediate intervention, and the approach can be individualized based on the specific circumstances. Here are general considerations:  Asymptomatic Pseudocysts: Observation: Small, asymptomatic pseudocysts may be observed without immediate intervention. Regular imaging and clinical monitoring are typically employed to assess for any changes or complications over time.  Indicatio3ns for Intervention: Intervention may be considered if the pseudocyst becomes symptomatic, enlarges significantly, or is associated with complications such as infection, bleeding, or compression of adjacent structures.  Pancreatic Necrosis:  Observation: Asymptomatic pancreatic necrosis may be initially managed conservatively without intervention, especially if it is not associated with systemic complications.  Indications for Intervention: Intervention, such as drainage or necrosectomy, may be warranted if there is evidence of infection within the necrotic tissue or if the necrosis is causing significant clinical complications.  Extrapancreatic Necrosis:  Observation: Similar to pancreatic necrosis, asymptomatic extrapancreatic necrosis may be observed without immediate intervention in the absence of complications.  Indications for Intervention: Intervention may be considered if extrapancreatic necrosis leads to complications such as infection, vascular compromise, or other issues affecting adjacent structures or organs.  Multidisciplinary Approach:  Case-by-Case Evaluation: The decision to intervene in cases of asymptomatic pseudocysts or necrosis often involves a multidisciplinary approach. Gastroenterologists, surgeons, and radiologists may collaborate to determine the most appropriate course of action based on the individual patient's condition.  Imaging Studies:  Regular Monitoring: Periodic imaging studies, such as contrast-enhanced computed tomography (CT) scans or magnetic resonance imaging (MRI), are essential for monitoring changes in pseudocysts or necrosis over time.  Response 2: The management of asymptomatic pseudocysts and pancreatic or extrapancreatic necrosis can vary depending on several factors, and decisions are typically made on a case-by-case basis. Here are some general considerations:  Asymptomatic Pseudocysts:  Pseudocysts are collections of fluid containing pancreatic enzymes, typically surrounded by a fibrous wall. If a pseudocyst is asymptomatic and not causing complications, immediate intervention may not be necessary. Observation with regular imaging to monitor the size and changes may be a reasonable approach.  Pancreatic Necrosis:  Pancreatic necrosis refers to dead tissue within the pancreas. Asymptomatic pancreatic necrosis may not always require immediate intervention. Management decisions depend on the extent of necrosis, the presence of symptoms, and the risk of complications. In some cases, a conservative approach with supportive care and monitoring may be appropriate.  Extrapancreatic Necrosis:  Similar to pancreatic necrosis, extrapancreatic necrosis may or may not require immediate intervention. The decision is influenced by factors such as the location and extent of necrosis, the presence of symptoms, and the risk of complications.  Complications and Symptoms:  If asymptomatic pseudocysts or necrosis become symptomatic or if complications such as infection, bleeding, or obstruction arise, intervention may be necessary. Symptoms may include abdominal pain, fever, jaundice, or signs of systemic illness.  Size and Stability:  The size and stability of pseudocysts or necrotic areas can also influence management decisions. Smaller, stable pseudocysts or necrotic areas may be managed conservatively, while larger or unstable collections may require intervention.  Intervention Options:  Interventions may include drainage procedures (such as percutaneous drainage, endoscopic drainage, or surgical drainage) or, in some cases, surgery to remove necrotic tissue. | Response 1: The presence of asymptomatic pseudocysts and pancreatic and/or extrapancreatic necrosis does not necessarily require intervention. The management of these conditions is typically guided by the following principles:  Asymptomatic Pseudocysts:  These are fluid collections that can occur as a consequence of acute pancreatitis. If they are small, not causing any symptoms, and not increasing in size on sequential imaging, they may be managed with observation alone. Intervention may be considered if a pseudocyst is large, persistent (usually present for more than six weeks), or causing symptoms such as abdominal pain, nausea, vomiting, or gastric outlet obstruction. Complications such as bleeding into the cyst or infection are also indications for intervention.  Pancreatic and/or Extrapancreatic Necrosis:  Sterile necrosis, even when extensive, is often managed conservatively without intervention, particularly in asymptomatic patients.  Intervention may become necessary if there are signs of infection such as fever, leukocytosis, or gas within the necrotic tissue on imaging, or if the necrosis is causing other complications like persistent pain or gastric outlet obstruction. The current trend in management is to delay intervention until the necrotic tissue has become well demarcated, often several weeks after the initial episode, as this can make the necrosectomy less challenging and potentially less risky.  Response 2: The management of asymptomatic pancreatic pseudocysts and pancreatic and/or extrapancreatic necrosis largely depends on their size, duration, and the presence of complications. In many cases, conservative management is preferred, especially for asymptomatic lesions. Here’s a guideline based on the current understanding and practices:  Asymptomatic Pseudocysts:  Intervention Not Routinely Required: Asymptomatic pseudocysts, especially if they are stable or decreasing in size, often do not require intervention. Many pseudocysts resolve spontaneously, particularly those less than 6 cm in size and present for less than six weeks.  Observation: Regular monitoring with imaging studies (e.g., ultrasound, CT scan) is recommended to assess for changes in size or the development of symptoms or complications.  Consider Intervention if: Intervention may be considered if the pseudocyst is large (typically >6 cm), persists for more than six weeks, or becomes symptomatic, or if complications develop (e.g., infection, rupture, or obstruction of adjacent organs).  Pancreatic and/or Extrapancreatic Necrosis:  Conservative Management for Asymptomatic Cases: Asymptomatic sterile necrosis often does not require intervention. Conservative management includes observation, supportive care, and nutritional support.  Intervention Criteria: Intervention may be considered in the presence of infected necrosis, symptomatic sterile necrosis (e.g., persistent pain, gastric outlet obstruction), or necrosis associated with complications that do not resolve with conservative management.  Timing and Approach: The timing of intervention for necrosis is crucial; delayed intervention (typically 4 weeks or more from onset), allowing for the liquefaction of necrosis and formation of a well-defined wall, is associated with better outcomes. Minimally invasive techniques (e.g., endoscopic, percutaneous, or laparoscopic approaches) are preferred over open surgery due to lower morbidity and mortality rates.  General Considerations:  Individualized Decision Making: The decision to intervene should be tailored to the individual patient's clinical scenario, taking into account the size and location of the pseudocyst or necrosis, the presence of symptoms, the patient's overall health and comorbidities, and the potential risks and benefits of intervention.  Multidisciplinary Approach: Management decisions should ideally involve a multidisciplinary team, including gastroenterologists, surgeons, and radiologists, to ensure a comprehensive approach to care.  In summary, asymptomatic pancreatic pseudocysts and necrosis do not routinely require intervention unless specific criteria are met, such as significant size, persistence, symptomatology, or complications. Regular monitoring and a tailored, patient-centered approach are essential components of management. | The presence of asymptomatic pseudocysts and pancreatic and/or extrapancreatic necrosis do not warrant intervention, regardless of size, location, and/or extension. |
| Is maintaining enteral nutrition thought to be helpful for patients with acute pancreatitis? | Response 1: Yes, maintaining enteral nutrition is considered beneficial for patients with acute pancreatitis. Enteral nutrition involves providing nutrition through the gastrointestinal (GI) tract, either orally or via a feeding tube, and it is generally preferred over parenteral nutrition (nutrition delivered intravenously) in the management of acute pancreatitis.  Key considerations supporting enteral nutrition in acute pancreatitis include:  Preservation of Gut Integrity: Enteral nutrition helps preserve the integrity of the gut mucosa and maintains the structural and functional integrity of the gastrointestinal tract. This is in contrast to parenteral nutrition, which bypasses the GI tract.  Reduced Risk of Infections: Enteral nutrition has been associated with a lower risk of infectious complications compared to parenteral nutrition. Preserving the gut barrier function helps prevent bacterial translocation and reduces the risk of infections, particularly in cases of severe pancreatitis.  Mitigation of Systemic Inflammatory Response: Enteral nutrition has been shown to mitigate the systemic inflammatory response associated with acute pancreatitis. It helps modulate the inflammatory response and promotes a more balanced immune response.  Early Initiation in Mild to Moderate Pancreatitis: In mild to moderate cases of acute pancreatitis, early initiation of enteral nutrition is often recommended. This can be started within 24 to 48 hours after admission, as tolerated by the patient.  Use of Elemental or Semi-Elemental Formulas: Elemental or semi-elemental enteral formulas may be preferred initially, as they are easier to absorb and may be better tolerated by the inflamed pancreas.  Avoidance of Oral Feeding in Severe Cases: In severe cases of acute pancreatitis with significant inflammation and systemic complications, oral feeding may be avoided initially, and enteral nutrition through a feeding tube may be preferred.  Individualized Approach: The approach to enteral nutrition should be individualized based on the patient's clinical condition, nutritional status, tolerance, and the severity of pancreatitis.  Continuous Monitoring: Continuous monitoring of the patient's nutritional status, laboratory parameters, and clinical response is important to adjust the enteral nutrition plan as needed.  Response 2: Yes, maintaining enteral nutrition is generally considered beneficial for patients with acute pancreatitis. Enteral nutrition involves providing nutrients directly into the gastrointestinal tract, often through a feeding tube, and it is preferred over parenteral nutrition (nutrient delivery through intravenous methods) in most cases of acute pancreatitis.  Here are several reasons why enteral nutrition is recommended:  Preservation of Gut Function:  Enteral nutrition helps to maintain the function of the gastrointestinal tract, preventing gut atrophy and promoting mucosal integrity. This is important for the overall digestive process.  Reduced Risk of Infections:  Enteral nutrition has been associated with a lower risk of infectious complications compared to parenteral nutrition. It helps to preserve the gut barrier and reduce bacterial translocation, which is the passage of bacteria from the gut to other parts of the body.  Immune Function Support:  Enteral nutrition supports the immune system, which is crucial for the body's defense against infections. It helps to maintain the gut-associated lymphoid tissue (GALT) and immune cells within the intestinal mucosa.  Improved Clinical Outcomes:  Studies have suggested that early enteral nutrition in acute pancreatitis is associated with reduced complications, shorter hospital stays, and improved clinical outcomes compared to delayed or no nutrition support.  Better Tolerance and Absorption:  Enteral nutrition is generally well-tolerated and associated with better absorption of nutrients compared to parenteral nutrition. This is important for providing essential nutrients during the recovery phase.  Avoidance of Pancreatic Stimulation:  Enteral nutrition can be formulated to minimize pancreatic stimulation, which is important in acute pancreatitis to reduce the workload on the inflamed pancreas.  The timing and mode of enteral nutrition may vary based on the severity of pancreatitis and the patient's condition. In some cases, early initiation of enteral nutrition within 24-48 hours of admission may be appropriate, while in others, a more cautious approach may be taken. | Response 1: Yes, maintaining enteral nutrition is generally thought to be helpful for patients with acute pancreatitis. Early enteral nutrition, which involves providing adequate nutrition through the gastrointestinal tract using either oral intake or tube feeding, is recommended in most cases.  Enteral nutrition helps to preserve gut barrier function, maintain intestinal motility, prevent bacterial translocation and infection, and improve overall outcomes. It is associated with reduced complications, shorter hospital stays, and improved mortality rates compared to parenteral nutrition (intravenous nutrition).  Enteral nutrition can be initiated within 48 hours of admission, even in patients with severe acute pancreatitis, as long as there are no contraindications such as intestinal obstruction or ileus.  The optimal composition of enteral nutrition for acute pancreatitis is still an area of ongoing research. However, a combination of polymeric formulas or blended regular diets with sufficient protein, carbohydrates, and fats is commonly used.  Response 2: Yes, maintaining enteral nutrition is considered beneficial for patients with acute pancreatitis, especially for those with moderate to severe disease. The approach to nutritional support in acute pancreatitis has evolved significantly over the years, with current evidence and guidelines supporting the use of enteral nutrition over parenteral nutrition whenever possible. Here are the key reasons why enteral nutrition is thought to be helpful in the management of acute pancreatitis:  Preservation of Gut Integrity: Enteral nutrition helps maintain the integrity and function of the gastrointestinal (GI) tract. It supports the mucosal barrier, reduces bacterial translocation from the gut, and thereby decreases the risk of infection.  Modulation of Inflammatory Response: Feeding through the GI tract can modulate the systemic inflammatory response and potentially reduce the severity of the inflammatory process in acute pancreatitis.  Prevention of Organ Failure: Early enteral nutrition has been associated with a reduced risk of organ failure and other complications in acute pancreatitis.  Reduction in Mortality and Complications: Several studies have shown that enteral nutrition can reduce mortality, infectious complications, and the length of hospital stay in patients with acute pancreatitis.  Feasibility and Safety: Enteral nutrition can often be administered safely even in severe cases of pancreatitis, using nasogastric or nasojejunal tubes if necessary. This is contrary to earlier concerns that feeding might stimulate the pancreas and worsen the condition. | Maintaining enteral nutrition is thought to help protect the gut, mucosal barrier and reduce bacterial translocation, thereby reducing the risk of infected peripancreatic necrosis and other serious acute pancreatitis outcomes |
| **Prevention** |  |  |  |
| What are the most common causes of acute pancreatitis? | Response 1: Acute pancreatitis can have various causes, and the most common ones include:  Gallstones:  Gallstones are a leading cause of acute pancreatitis. Gallstones can block the pancreatic duct, leading to inflammation.  Alcohol Consumption: xcessive alcohol intake is another major cause. Chronic alcohol abuse can contribute to the development of acute pancreatitis.  Idiopathic (Unknown Cause):  In some cases, the cause of acute pancreatitis remains unknown, and it is termed idiopathic. It may be related to factors not readily identified or specific genetic predispositions.  Trauma:  Physical trauma, such as abdominal injuries or surgery, can trigger pancreatitis.  Hypertriglyceridemia:  Elevated levels of triglycerides in the blood, known as hypertriglyceridemia, can cause acute pancreatitis.  Hypercalcemia:  High levels of calcium in the blood (hypercalcemia) can be associated with pancreatitis.  Infections:  Infections, particularly viral infections like mumps, can occasionally lead to acute pancreatitis.  Medications:  Certain medications, such as certain antibiotics, steroids, and immunosuppressants, have been linked to pancreatitis.  ERCP (Endoscopic Retrograde Cholangiopancreatography): ERCP is a procedure used for diagnostic and therapeutic purposes. While it is generally safe, it can, in some cases, lead to pancreatitis as a complication.  Autoimmune Pancreatitis:  An autoimmune reaction targeting the pancreas can lead to inflammation and pancreatitis.  Response 2: Acute pancreatitis is inflammation of the pancreas that occurs suddenly and is characterized by abdominal pain. The most common causes of acute pancreatitis include:  Gallstones:  Gallstones are a leading cause of acute pancreatitis. The stones can block the pancreatic duct, leading to inflammation. This is particularly common when a stone moves from the gallbladder into the common bile duct.  Alcohol Consumption:  Excessive alcohol intake is a significant risk factor for pancreatitis. Alcohol-induced pancreatitis can result from long-term heavy drinking or binge drinking.  Trauma or Injury:  Trauma to the abdomen, such as a car accident or a fall, can cause injury to the pancreas and trigger inflammation.  Hypertriglyceridemia:  Elevated levels of triglycerides (a type of fat) in the blood, known as hypertriglyceridemia, can lead to acute pancreatitis.  Infections:  Viral or bacterial infections, such as mumps or certain parasitic infections, may cause pancreatitis.  Medications:  Some medications, such as certain antibiotics, immunosuppressants, and diuretics, have been associated with an increased risk of pancreatitis.  ERCP (Endoscopic Retrograde Cholangiopancreatography):  This medical procedure, used to diagnose and treat conditions of the bile and pancreatic ducts, can sometimes lead to acute pancreatitis as a complication.  Autoimmune Conditions:  Autoimmune pancreatitis, where the body's immune system mistakenly attacks the pancreas, is a rare cause of pancreatitis.  Hereditary Factors:  In some cases, there may be a genetic predisposition to developing pancreatitis. | Response 1: The most common causes of acute pancreatitis include:  Gallstones: Gallstones that block the common bile duct can cause inflammation of the pancreas. This is the leading cause of acute pancreatitis in many people, especially women.  Alcohol consumption: Chronic and heavy alcohol use can lead to pancreatitis. It is one of the most common causes in men.  Trauma or injury: Physical trauma, such as a car accident or a blow to the abdomen, can damage the pancreas and trigger inflammation.  Medications: Certain medications, such as corticosteroids, diuretics, and some antibiotics, can cause pancreatitis in some individuals.  High triglyceride levels: Extremely high levels of triglycerides (a type of fat) in the blood can lead to pancreatitis.  ERCP (Endoscopic Retrograde Cholangiopancreatography): This procedure, used to diagnose and treat conditions of the bile and pancreatic ducts, can cause pancreatitis in rare cases.  Viral infections: Viral infections, especially mumps, can cause pancreatitis, although it is relatively rare.  Hereditary factors: Certain genetic disorders, such as hereditary pancreatitis and cystic fibrosis, can increase the risk of developing pancreatitis.  Response 2: The most common causes of acute pancreatitis are gallstones and alcohol consumption. These two factors account for the majority of cases in most populations. Other causes, while less common, can include a variety of conditions and factors:  Gallstones (Cholelithiasis): Gallstones can block the pancreatic duct, leading to pancreatitis. This obstruction causes pancreatic enzymes to become trapped in the pancreas, where they start digesting pancreatic tissue, causing inflammation and pain.  Alcohol Consumption: Chronic heavy alcohol use is a significant risk factor for acute pancreatitis. Alcohol can induce pancreatic inflammation directly and through the creation of protein plugs within the pancreatic duct.  Hypertriglyceridemia: Extremely high levels of triglycerides in the blood (typically above 1,000 mg/dL) can lead to acute pancreatitis.  Medications: Some medications have been associated with acute pancreatitis, though this is less common. Examples include certain diuretics, antiretrovirals, and immunosuppressive drugs.  Idiopathic: In a notable percentage of cases, the cause of acute pancreatitis remains unknown (idiopathic).  Genetic Factors: Certain genetic mutations can increase the risk of developing pancreatitis.  Autoimmune Pancreatitis: A rare form of pancreatitis caused by an autoimmune response.  Trauma: Physical injury to the abdomen can lead to pancreatitis, especially in children.  Infections: Certain viral, bacterial, and parasitic infections can cause acute pancreatitis. Viruses such as mumps, Coxsackie B, and hepatitis B are known examples.  Metabolic Disorders: Conditions such as hypercalcemia (high levels of calcium in the blood) can cause pancreatitis.  Surgical Procedures and Medical Interventions: Procedures such as endoscopic retrograde cholangiopancreatography (ERCP) can lead to pancreatitis as a complication. | The most common causes of acute pancreatitis remain gallstones and alcohol, which together comprise 80% of cases; the remainder of cases are due to less common causes, including drug reactions, pancreatic solid and cystic malignancies, and hypertriglyceridemia. |
| What are the well-studied interventions for patients undergoing a therapeutic ERCP to decrease the risk of post-ERCP pancreatitis, especially severe disease? | Response 1: Post-ERCP (Endoscopic Retrograde Cholangiopancreatography) pancreatitis is a known complication of the procedure, and efforts are made to minimize its occurrence. Several interventions have been studied to decrease the risk of post-ERCP pancreatitis, especially severe disease. Here are some well-studied interventions:  Pancreatic Duct Stenting:  Indication: Pancreatic duct stenting involves placing a stent in the pancreatic duct during ERCP.  Benefits: Stenting may help prevent post-ERCP pancreatitis, especially in high-risk cases. It allows drainage of pancreatic secretions, reducing the risk of pancreatic duct obstruction.  Rectal Nonsteroidal Anti-Inflammatory Drugs (NSAIDs):  Indication: Administration of rectal NSAIDs, such as diclofenac or indomethacin, before or after ERCP.  Benefits: NSAIDs have been shown to reduce the incidence of post-ERCP pancreatitis, possibly through their anti-inflammatory effects.  Prophylactic Pancreatic Duct Stenting and NSAIDs Combination:  Indication: Combined use of pancreatic duct stenting and rectal NSAIDs.  Benefits: Studies suggest that the combination of these interventions may be more effective in reducing the risk of post-ERCP pancreatitis than either intervention alone.  Sphincterotomy Technique:  Indication: Modification of sphincterotomy technique during ERCP.  Benefits: Some studies have investigated the impact of sphincterotomy techniques (e.g., small sphincterotomy) on reducing the risk of pancreatitis. However, results have been variable.  Wire-Guided Cannulation:  Indication: The use of wire-guided cannulation during ERCP.  Benefits: This technique may reduce the risk of post-ERCP pancreatitis compared to contrast injection cannulation methods.  Selective Cannulation:  Indication: Selective cannulation of the common bile duct.  Benefits: Studies have suggested that selective cannulation of the common bile duct (avoiding cannulation attempts in the pancreatic duct) may decrease the risk of post-ERCP pancreatitis.  Trainee Involvement:  Indication: Involvement of trainees (less experienced endoscopists) in ERCP procedures.  Benefits: Some studies have suggested that involvement of trainees may be associated with a higher risk of post-ERCP pancreatitis. Increased experience and expertise may contribute to better outcomes.  Use of Cap-assisted Endoscopy:  Indication: The use of a cap at the tip of the endoscope during ERCP.  Benefits: Some studies have investigated whether cap-assisted endoscopy can reduce the risk of post-ERCP pancreatitis, but results have been inconclusive.  Response 2: Post-ERCP (Endoscopic Retrograde Cholangiopancreatography) pancreatitis is a known complication of the procedure. Several well-studied interventions have been investigated to decrease the risk of post-ERCP pancreatitis, particularly severe cases. Here are some of the commonly studied interventions:  Pancreatic Duct Stenting:  Placement of a temporary pancreatic stent during ERCP has been shown to reduce the risk of post-ERCP pancreatitis. The stent helps to maintain ductal patency, preventing ductal obstruction and subsequent inflammation.  Prophylactic Pancreatic Duct Sphincterotomy:  Prophylactic sphincterotomy (cutting the sphincter of Oddi) during ERCP has been studied as a preventive measure for post-ERCP pancreatitis. However, the use of prophylactic sphincterotomy is still a topic of debate, and its routine application is not universally accepted.  Rectal Indomethacin (Nonsteroidal Anti-Inflammatory Drug - NSAID):  Administration of rectal indomethacin before or after ERCP has been found to be effective in reducing the risk of post-ERCP pancreatitis. The exact mechanism is not fully understood, but it is believed to involve anti-inflammatory effects.  Aggressive Hydration:  Maintaining adequate hydration before and after the procedure has been suggested as a preventive measure. However, the evidence supporting the efficacy of aggressive hydration is variable.  Use of Low-Osmolar Contrast Media:  Some studies suggest that using low-osmolar contrast media during ERCP may decrease the risk of post-ERCP pancreatitis compared to high-osmolar contrast media.  Selective Cannulation Techniques:  The technique used for cannulation of the pancreatic duct or bile duct may influence the risk of post-ERCP pancreatitis. Strategies such as wire-guided cannulation and pancreatic guidewire placement have been studied.  Use of Cap-assisted Endoscopy:  Cap-assisted endoscopy, which involves attaching a soft cap to the endoscope, has been investigated as a potential technique to reduce the risk of post-ERCP pancreatitis.  It's important to note that the choice of interventions may depend on the individual patient's risk factors, anatomy, and the nature of the ERCP procedure. The decision to use specific preventive measures should be made by the treating physician based on a careful assessment of the patient's condition. | Response 1: Several interventions have been studied to decrease the risk of post-ERCP pancreatitis, especially severe disease. These interventions include:  Pancreatic stent placement: The placement of a temporary pancreatic stent during ERCP has been shown to significantly reduce the risk of post-ERCP pancreatitis, particularly in high-risk patients. The stent helps to prevent pancreatic duct obstruction and maintain the drainage of pancreatic secretions.  Rectal NSAIDs: The administration of nonsteroidal anti-inflammatory drugs (NSAIDs) like indomethacin or diclofenac rectally before or after ERCP has been found to be effective in reducing the incidence of post-ERCP pancreatitis, especially in high-risk patients.  Prophylactic pancreatic duct stenting: In selected patients at high risk for post-ERCP pancreatitis, prophylactic placement of a pancreatic duct stent, even in the absence of pancreas divisum or pancreatic ductal abnormalities, may be considered to mitigate the risk.  Use of secretin: Some studies have evaluated the use of secretin, a hormone that stimulates pancreatic secretion, to reduce the risk of post-ERCP pancreatitis. However, the results have been conflicting, and further research is needed to establish its effectiveness.  Guidewire cannulation techniques: Certain techniques used during ERCP, such as wire-guided cannulation and the use of pancreatic duct guide wires, have been associated with a lower risk of post-ERCP pancreatitis compared to conventional cannulation methods. These techniques aim to minimize trauma to the pancreatic duct during the procedure.  Response 2: Several interventions have been studied and implemented to decrease the risk of post-endoscopic retrograde cholangiopancreatography (ERCP) pancreatitis (PEP), especially severe disease. These interventions focus on identifying high-risk patients, using procedural techniques to minimize pancreatic duct injury, and pharmacological measures. Some of the most well-studied and effective interventions include:  Rectal NSAIDs: The administration of rectal non-steroidal anti-inflammatory drugs (NSAIDs), particularly indomethacin or diclofenac, immediately before or after ERCP, has been shown to significantly reduce the incidence of PEP. This intervention is recommended for both high-risk and average-risk patients undergoing ERCP.  Pancreatic Duct Stenting: Placement of a short, temporary stent in the pancreatic duct during ERCP can reduce the incidence of PEP in high-risk patients. This technique is thought to facilitate pancreatic drainage, reducing ductal pressure and inflammation.  Aggressive Hydration: Adequate hydration, particularly with lactated Ringer’s solution, before and after the procedure, has been suggested to reduce PEP risk. This intervention aims to improve pancreatic microcirculation and reduce the effects of toxic metabolites.  Selective Cannulation Techniques: Using advanced cannulation techniques and devices, such as guidewire-assisted cannulation, to minimize trauma to the pancreatic duct can reduce the incidence of PEP. Avoiding multiple attempts at cannulation and minimizing manipulation of the pancreatic duct are key strategies.  Prophylactic Pharmacotherapy (other than NSAIDs): Although various pharmacological agents have been studied (e.g., protease inhibitors, nitrates, calcium channel blockers), most have not shown consistent benefit in preventing PEP. Currently, rectal NSAIDs are the most widely supported pharmacological intervention.  Endoscopic Technique Modification: Techniques such as avoiding precut sphincterotomy when possible and minimizing pancreatic duct injection can reduce PEP risk. The use of intraductal ultrasound instead of contrast injection into the pancreatic duct for certain diagnostic purposes may also reduce PEP incidence.  Patient Selection: Careful selection of patients for ERCP, ensuring the procedure is indicated and considering alternative diagnostic or therapeutic approaches for patients at high risk for PEP, is crucial.  Training and Experience: ERCPs performed by experienced endoscopists with high procedural volumes are associated with lower rates of PEP. Continuous training and adherence to best practices are important for minimizing complications. | For patients undergoing a therapeutic ERCP, three well-studied interventions to decrease the risk of post-ERCP pancreatitis, especially severe disease:(i) guidewire cannulation,(ii) pancreatic duct stents, and (iii) rectal NSAIDs. |
| **Others** |  |  |  |
| What is the definition of idiopathic acute pancreatitis? | Response 1: Idiopathic acute pancreatitis refers to cases of pancreatitis in which the cause or underlying etiology cannot be identified despite thorough investigation. In other words, it is pancreatitis that occurs without a clear and identifiable cause based on available clinical, laboratory, and imaging assessments. The term "idiopathic" is used when the origin or trigger of the pancreatitis remains unknown.  The diagnosis of idiopathic acute pancreatitis is typically made after a comprehensive evaluation that includes:  Clinical Assessment: Detailed history taking, including information about alcohol consumption, recent medications, dietary habits, and the presence of any underlying medical conditions.  Laboratory Tests: Blood tests to assess pancreatic enzyme levels (amylase and lipase) and identify potential causes such as hypertriglyceridemia or hypercalcemia.  Imaging Studies: Radiological investigations, such as abdominal ultrasound, computed tomography (CT) scan, or magnetic resonance imaging (MRI), to evaluate the pancreas and surrounding structures for abnormalities.  Endoscopic Retrograde Cholangiopancreatography (ERCP) or Magnetic Resonance Cholangiopancreatography (MRCP): In some cases, more invasive procedures like ERCP or non-invasive imaging like MRCP may be performed to visualize the pancreatic and bile ducts.  Response 2: Idiopathic acute pancreatitis refers to cases of acute pancreatitis where the underlying cause or trigger is unknown or cannot be identified despite a thorough investigation. In other words, "idiopathic" means that the cause of the condition is not readily apparent or has not been determined.  Acute pancreatitis is characterized by the sudden inflammation of the pancreas, leading to symptoms such as severe abdominal pain, nausea, and vomiting. Common known causes of acute pancreatitis include gallstones, alcohol consumption, certain medications, infections, and structural abnormalities of the pancreas or biliary system.  When a thorough evaluation, including medical history, laboratory tests, imaging studies, and other diagnostic procedures, fails to reveal a specific cause for the pancreatitis episode, it is classified as idiopathic. Idiopathic acute pancreatitis can be challenging for both patients and healthcare providers because the absence of a clear cause makes it difficult to implement targeted preventive measures.  In some cases, the cause of idiopathic acute pancreatitis may become apparent over time, or it may remain unknown. Management often involves supportive care, addressing symptoms, and monitoring for potential recurrence. If a patient experiences repeated episodes of idiopathic acute pancreatitis, further evaluation and consultation with specialists may be pursued to identify any underlying factors or contributing conditions that were initially missed. | Response 1: Idiopathic acute pancreatitis refers to a condition where the cause of pancreatitis cannot be identified despite a thorough evaluation. It is a diagnosis of exclusion, meaning that other identifiable causes of pancreatitis, such as gallstones or alcohol use, have been ruled out through medical history, physical examination, laboratory tests, and imaging studies.  It is estimated that approximately 10-15% of acute pancreatitis cases are classified as idiopathic, where no specific cause can be determined. The exact underlying mechanisms of idiopathic acute pancreatitis are not fully understood, but it is believed that a combination of genetic, environmental, and other factors may contribute to its development.  In cases of idiopathic pancreatitis, management focuses on supportive care, alleviating symptoms, and preventing further attacks. Additionally, lifestyle modifications, such as reducing alcohol consumption, maintaining a healthy weight, and following a balanced diet, are generally recommended to reduce the risk of recurrent episodes.  It is important to note that while the initial episode may be classified as idiopathic, further investigations and monitoring may be warranted in some cases to identify any underlying causes that were initially missed, as recurrent pancreatitis episodes may provide additional clues for diagnosis.  Response 2: Idiopathic acute pancreatitis refers to cases of acute pancreatitis in which no obvious cause can be identified after a comprehensive evaluation. Despite thorough history taking, physical examination, laboratory testing, imaging studies, and sometimes more advanced diagnostic procedures, the etiology of the pancreatitis remains unknown in these patients. Common causes that are typically investigated and ruled out include gallstones, alcohol use, medications, hypertriglyceridemia, and anatomical abnormalities of the pancreatic and biliary ducts.  The diagnostic workup for idiopathic acute pancreatitis often includes:  Blood tests: To check for elevated levels of pancreatic enzymes (amylase and lipase), liver function tests, lipid profile, and to screen for infections or other systemic causes.  Imaging studies: Such as abdominal ultrasound, computed tomography (CT) scan, magnetic resonance imaging (MRI), and magnetic resonance cholangiopancreatography (MRCP) to look for gallstones, structural abnormalities, and signs of chronic pancreatitis.  Endoscopic evaluation: Endoscopic ultrasound (EUS) or endoscopic retrograde cholangiopancreatography (ERCP) might be considered in some cases to investigate for microlithiasis (tiny gallstones), subtle anatomical abnormalities, or other causes not visible on non-invasive imaging. | Idiopathic acute pancreatitis is defined as pancreatitis with no etiology established after initial laboratory (including lipid and calcium level) and imaging tests (transabdominal ultrasound and CT in the appropriate patient). |
| Do we need more evidence to optimize the management of acute pancreatitis? | Response 1: While there has been significant progress in understanding and managing acute pancreatitis, ongoing research and the generation of additional evidence are crucial to continually optimize its management. Acute pancreatitis is a complex condition with varying presentations and potential complications, and there are several areas where more evidence and research are needed to refine treatment strategies. Here are some aspects where additional evidence could contribute to optimizing the management of acute pancreatitis:  Identification of Biomarkers: More research is needed to identify reliable biomarkers that can aid in the early diagnosis, severity assessment, and prognosis of acute pancreatitis. Improved biomarkers could enhance risk stratification and guide personalized treatment approaches.  Optimal Timing of Interventions: The timing of interventions, such as cholecystectomy or endoscopic procedures, remains an area of ongoing research. Determining the optimal timing for specific interventions can impact outcomes and reduce complications.  Nutritional Support Strategies: While enteral nutrition is generally recommended, further research could refine the specific nutritional support strategies, including the timing, composition, and duration of enteral nutrition in different subgroups of patients.  Prevention of Recurrent Pancreatitis: Understanding the factors contributing to recurrent acute pancreatitis and developing targeted interventions for prevention are areas where more evidence could lead to improved outcomes.  Pharmacological Therapies: Exploring and validating the effectiveness of pharmacological therapies, including anti-inflammatory agents or other medications targeting specific pathways, could offer additional treatment options.  Genetic and Molecular Insights: Investigating the genetic and molecular basis of acute pancreatitis may help identify predisposing factors, clarify disease mechanisms, and guide personalized treatment approaches.  Optimal Use of Imaging Techniques: Further research on the optimal use and timing of imaging techniques, such as contrast-enhanced CT scans or MRI, can contribute to improved diagnostic accuracy and monitoring of the disease course.  Risk Prediction Models: Developing and validating accurate risk prediction models for acute pancreatitis could assist in early identification of patients at higher risk for complications, allowing for more targeted interventions and resource allocation.  Comparative Effectiveness Research: Comparative effectiveness research comparing different treatment modalities, including endoscopic interventions, surgery, and medical management, in specific patient populations could guide evidence-based decision-making.  Patient-reported Outcomes: Assessing and incorporating patient-reported outcomes in clinical trials can provide valuable insights into the impact of acute pancreatitis and its management on patients' quality of life.  Response 2: The field of acute pancreatitis continues to be an area of active research, and there is an ongoing effort to gather more evidence to optimize its management. While significant progress has been made in understanding the condition and refining treatment strategies, there are still areas where additional research could contribute to improved patient outcomes. Some aspects where more evidence might be beneficial include:  Risk Stratification:  Identifying better methods for risk stratification to predict the severity of acute pancreatitis early on. This could help tailor treatment approaches based on the individual patient's risk profile.  Nutritional Support:  Further research on the optimal timing, route, and composition of nutritional support in acute pancreatitis, considering the balance between early enteral nutrition and the risk of exacerbating inflammation.  Fluid Management:  Clarifying the best approach to fluid resuscitation and management during the early stages of acute pancreatitis, taking into account individual patient factors and the risk of complications.  Interventions for Complications:  Evaluating the effectiveness of different interventions for managing complications such as pseudocysts, pancreatic necrosis, and infected necrosis. This includes exploring the timing and type of interventions.  Prevention of Recurrent Pancreatitis:  Investigating strategies for preventing recurrent episodes of pancreatitis in individuals with a history of the condition, especially in cases where the cause remains elusive.  Role of Novel Therapies:  Exploring the potential benefits of novel therapies or medications in the treatment of acute pancreatitis, including anti-inflammatory agents or other pharmacological interventions.  Genetic and Molecular Factors:  Understanding the genetic and molecular factors that contribute to the development and progression of acute pancreatitis. This could lead to more personalized and targeted treatment approaches.  Patient-Centered Outcomes:  Assessing patient-centered outcomes and quality of life in individuals who have experienced acute pancreatitis to guide long-term management strategies. | Response 1: As an AI assistant, I can provide some insights on this topic. While the existing evidence base for the management of acute pancreatitis is substantial, ongoing research is still valuable for optimizing its management. There are several reasons why more evidence is necessary:  Evolving understanding: Medical knowledge and understanding are constantly evolving. New research can help update guidelines and recommendations based on the latest evidence, ensuring that healthcare professionals are equipped with the most current information for managing acute pancreatitis.  Individualized treatment: Acute pancreatitis is a complex condition that can present in various ways and have different causes and outcomes. Further research can help identify specific subgroups within the patient population who may benefit from tailored treatment approaches, such as those with severe pancreatitis or specific etiologies like alcoholic pancreatitis.  Novel interventions: Ongoing research can explore and evaluate new therapies, procedures, or interventions for acute pancreatitis that may improve patient outcomes. This includes investigating potential medications, surgical techniques, or minimally invasive procedures that may offer advancements in the management of the condition.  Long-term consequences: Acute pancreatitis can have long-term consequences, including the development of chronic pancreatitis or recurrent episodes. Further research on long-term follow-up and outcomes can provide insights into optimizing post-acute management strategies and preventing long-term complications.  Cost-effectiveness: Assessing the cost-effectiveness of different management strategies is crucial for healthcare resource allocation. Continued research can help identify strategies that provide the best outcomes while also considering the economic impact on healthcare systems and patients.  Patient-centered care: Understanding the impact of acute pancreatitis on patients' quality of life and well-being is essential. Research focusing on patient-reported outcomes can guide the development of patient-centered management approaches, ensuring that interventions not only improve clinical outcomes but also enhance patients' overall experience and satisfaction.  In conclusion, while the existing evidence base for acute pancreatitis management is substantial, ongoing research is still necessary to optimize its management. By further refining treatment approaches, evaluating novel interventions, considering long-term consequences, assessing cost-effectiveness, and focusing on patient-centered care, we can continually improve patient outcomes in the management of acute pancreatitis.  Response 2: Yes, more evidence is needed to optimize the management of acute pancreatitis. While significant advances have been made in understanding and treating acute pancreatitis, several areas require further research to improve patient outcomes. Some of these areas include:  Early Identification and Risk Stratification: Developing more accurate and early predictors of disease severity could improve initial management strategies and patient triage, potentially reducing morbidity and mortality.  Fluid Management: Optimal fluid therapy, including the type of fluid, rate of administration, and timing, remains an area of ongoing debate. More evidence is needed to establish guidelines that can be personalized to patient needs.  Nutritional Support: The timing, route (enteral vs. parenteral), and composition of nutritional support for different severities of acute pancreatitis need further clarification to enhance recovery and minimize complications.  Management of Complications: Research into the best practices for managing complications of acute pancreatitis, such as necrotizing pancreatitis, infected necrosis, and pseudocysts, is ongoing. This includes the timing and approach to intervention (endoscopic, percutaneous, or surgical) and the management of systemic complications.  Role of ERCP: The timing and indications for ERCP in the management of gallstone pancreatitis and its role in preventing recurrent episodes need further clarification, especially in mild cases.  Pharmacological Interventions: Despite numerous trials, no specific pharmacological treatment has been proven effective in altering the disease course of acute pancreatitis. Research into new therapeutic agents, including those targeting the inflammatory cascade, is needed.  Genetic and Environmental Factors: Understanding the role of genetic predispositions and environmental factors in the development and progression of acute pancreatitis could lead to personalized prevention strategies and treatments.  Long-term Outcomes: More studies are needed on the long-term outcomes of acute pancreatitis, including the risk of recurrent attacks, chronic pancreatitis development, and the impact on quality of life.  Prevention: Identifying effective strategies for preventing acute pancreatitis, particularly in high-risk populations, remains a crucial research area.  Guideline Implementation: Evaluating the best strategies for implementing evidence-based guidelines into clinical practice to ensure that all patients receive optimal care is essential. | There are several knowledge gaps in the initial management of acute pancreatitis that have been identified for which RCTs are warranted, as is highlighted in the technical review that accompanies this guideline. |
