# Supplementary material for: Exploring the performance of ChatGPT on acute pancreatitis-related questions
Source: J Transl Med. 2024 Jun 1;22:527. doi: 10.1186/s12967-024-05302-8 (PMC11143553; doi:10.1186/s12967-024-05302-8)
Supplement: Supplementary file 4 — Supplementary Material 4. [file 12967_2024_5302_MOESM4_ESM.docx]

| **Table S2 Examples of medical licensing examination test questions and the performance of ChatGPT.** | | | | | | | | | |
| --- | --- | --- | --- | --- | --- | --- | --- | --- | --- |
| Test questions by Subfields | Options | | | | | standard answer | Answers from ChatGPT 3.5 | Answers from ChatGPT 4.0 | Accuracy from examinees |
|  | A | B | C | D | E |  |  |  |  |
| **Diagnosis** |  |  |  |  |  |  |  |  |  |
| Hemorrhagic necrotic acute pancreatitis may have | Elevated serum amylase | Serum amylase is normal or lower than normal | both | Neither |  | C | A,C | C,C | 0.5044 |
| The most valuable diagnostic tool for hemorrhagic necrotizing pancreatitis is | Increased blood lipase | Increased blood amylase | Decreased blood calcium | Increased blood bilirubin | B-ultrasound examination of pancreatic enlargement | C | B,B | C,C | 0.4743 |
| Regarding the changes in serum amylase during acute pancreatitis, the incorrect description is | Begins to rise 2-12 hours after onset of symptoms | Blood amylase often exceeds 3 times the normal value | Urinary amylase remains elevated longer than blood amylase | The level of blood amylase value is directly proportional to the degree of disease | Urine amylase measurement lasts 1-2 weeks | D | E,C | D,D | 0.8355 |
| Patient, male, 40 years old. I started having severe pain in my upper abdomen 8 hours ago. At this time, the examination with little value for diagnosis is | Blood amylase measurement | Urine amylase assay | electrocardiogram | Plain abdominal X-ray |  | B | C,C | C,C | 0.5918 |
| Patient, male. A 45-year-old patient suffered from upper abdominal pain for 4 days accompanied by nausea and vomiting. Vomiting a small amount of gastric contents, physical examination revealed upper abdominal tenderness, suspicious jaundice of the sclera, body temperature of 38°C, white blood cells of 15X109L, serum amylase value of 64 Wen's units, and urinary amylase of 326 Wen's units. can be considered as | acute pancreatitis | acute cholecystitis | acute cholangitis | acute mesenteric lymphadenitis | ulcer perforation | A | A,A | A,A | 0.9675 |
| The most meaningful examination for acute hemorrhagic necrotizing pancreatitis is | plain abdominal radiograph | Abdominal B-ultrasound | Abdominal CT | Blood routine | Biochemical tests | C | C,C | C,C | 0.9009 |
| Laboratory examination characteristics of acute hemorrhagic necrotizing pancreatitis | Serum amylase was significantly increased | hyperbilirubinemia | Increased serum methemoalbumin | Increased amylase endogenous clearance rate ratio | Serum calcium significantly decreased | E | E,E | E,E | 0.6971 |
| One week after the onset of acute abdominal pain, the examination with more diagnostic value for pancreatitis is: | White blood cell count and classification | serum amylase | fasting blood glucose | serum lipase | urinary amylase | D | D,D | D,D | 0.4429 |
| In acute necrotizing pancreatitis, which of the following test results is correct? | Serum amylase was elevated | Early elevation of serum lipase | elevated blood sugar | Elevated blood calcium | Elevated blood albumin | C | A,C | C,C | 0.5185 |
| In acute pancreatitis, the pattern of elevated serum amylase is | Increased 2 hours after onset | 3 to 12 hours after onset | Begins to rise 24 hours after onset of symptoms | Begins to increase 48 hours after onset of symptoms | None of the above is correct | B | A,A | A,B | 0.4583 |
| Biochemical test errors for acute pancreatitis are | Persistent fasting blood glucose higher than 10mmol/L indicates a serious prognosis | Hypercholesterolemia may occur | Serum albumin reduces mortality and mortality | Hyperbilirubinemia may occur | Blood calcium lower than 1.75mmol/L is seen in hemorrhagic necrotizing pancreatitis | C | A,A | C,E | 0.697 |
| In patients with acute pancreatitis, if the high fever persists for 2 to 3 weeks and a mass can be palpated in the upper abdomen, suspicion should be raised first. | septicemia | suppurative cholangitis | subseptal abscess | pancreatic abscess | none | D | D,D | D,D | 0.9324 |
| In patients with acute pancreatitis, the following indicators indicate a poor prognosis, except which one | Abnormally elevated blood and urine amylase | Blood calcium dropped to 1.75mmol/L | Decreased serum albumin | Hypoxemia (arterial partial pressure of oxygen 7.98kPa) | long lasting shock | A | D,A | A,A | 0.6747 |
| The time for pseudocyst formation in acute pancreatitis is usually after the disease | 3~4 hours | 24 hours | 3~4 days | 3~4 weeks | March~April | D | D,D | D,D | 0.6776 |
| In acute pancreatitis, the correct statement about amylase measurement is | Only increased blood and urine amylase | Serum amylase peaks at 8 hours | Serum amylase more than 2 times normal can be diagnosed as acute pancreatitis | The level of amylase does not necessarily reflect the severity of the disease | Increased urinary amylase can last for 2 to 4 weeks | D | D,D | D,D | 0.8883 |
| In acute pancreatitis, the time when blood lipase begins to rise is usually after the onset of the disease | 72 hours | 24 hours | 48 hours | 10 hours | 2 hours | B | B,B | B,A | 0.4752 |
| The time when blood amylase rises in acute pancreatitis is usually after the symptoms appear. | immediately | 4 hours | 24 hours | 2-12 hours | When urinary amylase is elevated | D | D,D | D,D | 0.7831 |
| The time when pseudocyst forms in acute pancreatitis is | 1~2 days after illness | 2 to 3 weeks after illness | 3 to 4 weeks after illness | 5 to 6 weeks after illness | none | C | B,B | C,C | 0.5819 |
| Sudden death can occur | hemorrhagic necrotizing pancreatitis | latent myocardial infarction | both | neither |  | C | C,C | A,A | 0.8538 |
| Can damage other organs and cause shock due to | Activate elastic fiber enzyme | Activate phospholipase A | activate pancreatic vasodilator | Activate trypsin | Activate plasma skin tone | E | D,E | D,D | 0.243 |
| At present, the test indicators that are important for the early diagnosis of acute pancreatitis are | Blood and urine amylase | white blood cells | serum lipase | blood sugar | blood calcium | A | A,A | C,A | 0.9339 |
| Male, 30 years old. One day ago, I developed epigastric pain and vomiting after drinking a large amount of alcohol. The pain did not relieve after vomiting and worsened with abdominal distension for 3 hours. Blood amylase was 650U/L ( somogyi ), blood pressure was 80/60mmHg, pulse was 120 beats/min, and the skin around the umbilicus and both ribs and abdomen were purple. The most likely diagnosis is | acute severe pancreatitis | acute cholecystitis | acute gastritis | acute hepatitis | intestinal obstruction | A | A,A | A,A | 0.99 |
| 40-year-old man suffered from epigastric pain radiating to his left shoulder, waist, and back, nausea, vomiting, and abdominal distension starting 5 hours after dinner. It’s been 37 hours now. There was a history of gallstones. Physical examination showed: breathing 24 times/min, body temperature 38.9°C, and blood pressure 90/75mmHg. Suspicious jaundice of the sclera, tenderness throughout the abdomen, especially in the upper abdomen, accompanied by muscle tension and rebound tenderness, positive shifting dullness, white blood cells of 16X10°/, and neutrophils of 89%. To confirm the diagnosis, the most valuable tests are | Determination of blood amylase | Determination of urinary amylase | Abdominal puncture fluid examination and determination of amylase | abdominal ultrasound | Abdominal X-ray | C | C,C | C,D | 0.471 |
| Male, 40 years old. Persistent upper abdominal pain for 10 hours after drinking, radiating to the lower back, accompanied by nausea, vomiting, fever, and no hematuria. The most meaningful test for a definite diagnosis is | blood amylase | Blood routine | serum lipase | urinary amylase | Plain X-ray of abdomen | A | A,A | A,A | 0.8904 |
| Male, 40 years old. Persistent upper abdominal pain for 10 hours after drinking, radiating to the lower back, accompanied by nausea, vomiting, fever, and no hematuria. The most likely diagnosis is | acute pancreatitis | cholecystitis | peptic ulcer | intestinal obstruction | kidney stones | A | A,A | A,A | 0.9848 |
| A 45-year-old man developed mid-upper abdominal pain radiating to both waists 8 hours after drinking alcohol, accompanied by nausea and vomiting. The following checks should be selected first | Hematuria | Urine amylase assay | Plain X-ray of chest and abdomen | Serum amylase assay | Abdominal B-mode ultrasound | D | D,D | D,D | 0.8874 |
| Male, 45 years old. After eating a high-fat meal and drinking alcohol, the pain in the upper abdomen continued for 8 hours, and the pain was not relieved after vomiting twice. Check the left side of the upper abdomen for tenderness and rebound tenderness: T37.8°C. The most likely diagnosis is | acute gastritis | acute cholecystitis | intestinal obstruction | acute pancreatitis | acute myocardial infarction | D | D,D | D,D | 0.9798 |
| A 65-year-old man suffered from sudden epigastric pain for 1 day, fever for 6 hours, and dyspnea for 1 hour. Physical examination: T38.6C, R32 times/min, BP90/60mmHg, P110 times/min, mild scleral jaundice, upper abdominal tenderness, and mild Muscle tension. Bowel sounds 1 time/min. Blood amylase was 860U, blood oxygen partial pressure was 50mmHg. The first consideration to diagnose is | Acute cholangitis with infection and toxic shock | mesenteric artery embolism | pneumonia | acute myocardial infarction | Acute pancreatitis and respiratory distress syndrome | E | A,E | E,E | 0.9124 |
| Male, 41 years old. Upper abdominal pain for 7 hours. She was accompanied by fever with a body temperature of 38.5°C and frequent vomiting. Physical examination revealed upper abdominal muscle tension and tenderness without shifting dullness. White blood cells 15X10°/L. X-ray examination: no free gas was found under the septum. In order to confirm the diagnosis, the items that need to be checked urgently are: | blood amylase | Blood routine | serum lipase | urinary amylase | Urine routine | A | A,A | A,A | 0.9585 |
| Male, 41 years old. Upper abdominal pain for 7 hours. She was accompanied by fever with a body temperature of 38.5°C and frequent vomiting. Physical examination revealed upper abdominal muscle tension and tenderness without shifting dullness. White blood cells 15X10°/L. X-ray examination: no free gas was found under the septum. If a patient develops an upper abdominal mass during treatment, the first diagnosis to consider is | peritoneal metastasis cancer | Adhesion intestinal obstruction | pancreatic pseudocyst | pancreatic cancer | colon cancer | C | C,C | C,C | 0.8951 |
| Male, 41 years old. Upper abdominal pain for 7 hours. She was accompanied by fever with a body temperature of 38.5°C and frequent vomiting. Physical examination revealed upper abdominal muscle tension and tenderness without shifting dullness. White blood cells 15X10°/L. X-ray examination: no free gas was found under the septum. The most likely diagnosis is | acute myocardial infarction | acute pancreatitis | cholelithiasis | gastric ulcer perforation | intestinal obstruction | B | B,B | B,B | 0.98 |
| Male, 46 years old. After drinking alcohol, he developed persistent pain in the middle and upper abdomen for 24 hours, and he vomited twice. The vomitus was gastric contents. The abdominal pain did not relieve after vomiting, so he was admitted to the emergency hospital. To confirm the diagnosis, the most valuable examination is | Blood amylase measurement | Ascitic fluid amylase assay | Abdominal B-mode ultrasound | plain abdominal radiograph |  | B | C,C | A,C | 0.6789 |
| Male, 78 years old, vomiting, abdominal distension for 21 hours, no obvious abdominal pain, history of peptic ulcer, upper abdominal tenderness, abdominal muscle tension, blood pressure 80/50mmHg, pulse 108 beats/min, blood amylase 250U/L, blood calcium 1.7mmol/L The most likely diagnosis is | acute myocardial infarction | acute pancreatitis edema type | acute pancreatitis hemorrhagic necrotic type | acute intestinal obstruction | Acute perforation of peptic ulcer | C | E,E | E,B | 0.7563 |
| Female, 40 years old. He was diagnosed with acute pancreatitis, and the upper abdominal tenderness was obvious after 2 weeks of regular medical treatment. The body temperature is still 38°C-39°C, the blood amylase is 256U/L, and the blood WBC16X10%/L. The most likely thing is | septicemia | The illness is delayed and has not been cured | Acute cholecystitis | Complicated pancreatic pseudocyst | Complicated pancreatic abscess | E | B,E | E,E | 0.7312 |
| A 42-year-old female came to the doctor with sudden persistent pain in the upper abdomen accompanied by nausea and vomiting for 8 hours after attending a banquet. The most likely diagnosis was | acute intestinal obstruction | acute appendicitis | acute cholecystitis | acute pancreatitis | peptic ulcer perforation | D | C,C | D,D | 0.9718 |
| A 42-year-old female came to the doctor for 8 hours after attending a banquet with sudden persistent pain in the upper abdomen accompanied by nausea and vomiting. The preferred examination was | urinary amylase | blood amylase | blood lipase | Abdominal B-ultrasound | plain abdominal radiograph | B | D,D | D,D | 0.9223 |
| Female, 48 years old. After eating a large amount of meat, I suffered from epigastric pain and vomiting for 6 hours. The abdominal pain was persistent, worsened in episodes, and radiated to the left lower back. The vomitus was gastric contents. The most significant laboratory test for a definite diagnosis is | urinary amylase | blood amylase | blood white blood cell count | blood bilirubin | Urine routine | B | B,B | B,B | 0.9716 |
| Female, 54 years old. I have a history of gallbladder stones for 8 years. Severe pain in the upper abdomen for 2 days, radiating to the waist, accompanied by nausea and vomiting, and blood amylase increased by 2 times. The following most valuable examinations are | plain abdominal radiograph | Upper gastrointestinal barium meal | electrocardiogram | Abdominal CT | gastroscopy | D | D,D | D,D | 0.9341 |
| Edematous acute pancreatitis may have | Elevated serum amylase | Serum amylase is normal or lower than normal | Have both | Neither |  | A | B,B | C,A | 0.4132 |
| Which of the following imaging tests can be used as routine initial screening tests for acute pancreatitis? | plain abdominal radiograph | B-ultrasound | CT | MRI | none | B | B,B | B,B | 0.6363 |
| Among the following examinations related to acute pancreatitis, the one with the earliest abnormality is | serum lipase | serum methemoalbumin | serum amylase | urinary amylase | serum lactate dehydrogenase | C | C,C | C,C | 0.9471 |
| The most meaningful test for diagnosing acute hemorrhagic necrotizing pancreatitis is | Blood amylase test | Serum alpha-fetoprotein test | Abdominal CT examination | Blood CEA | Blood CRP | C | C,C | C,C | 0.5115 |
| The important basis for diagnosing acute pancreatitis is | Upper abdominal pain | Abdominal bloating, flatulence, and cessation of defecation | Diminished bowel sounds | Serum amylase exceeds 128 Wen units | Plain X-ray of abdomen | D | A,A | D,D | 0.8405 |
| **Clinical feture** |  |  |  |  |  |  |  |  |  |
| Which of the following statements about the clinical manifestations of acute pancreatitis is incorrect? | Abdominal pain can radiate to the lower back | There may be a lump in the upper abdomen | Abdominal signs consistent with severity of abdominal pain | Have mild fever | none | C | B,B | C,C | 0.6422 |
| Cullen's sign in hemorrhagic necrotizing pancreatitis | The flank skin is gray-purple spots | Gray-purple spots on the skin around the umbilicus | Blue and purple skin on flank | The skin around the umbilicus is blue and purple | Periumbilical skin erythema | D | B,D | B,B | 0.3546 |
| Which of the following is false regarding acute pancreatitis? | Hypercalcemia can cause pancreatic duct calcification, increase pancreatic juice secretion and promote trypsinogen activation | "Common channel" is the only theory explaining acute pancreatitis caused by biliary tract disease | Most of the digestive enzymes secreted by the normal pancreas are inactive zymogens. | Acute pancreatitis is a chemical inflammation of the pancreatic tissue itself due to digestion | Related to the reduction of physiological trypsin inhibitory substances | B | E,B | B,B | 0.7401 |
| Local complications of acute hemorrhagic necrotizing pancreatitis | Massive upper gastrointestinal bleeding | acute renal failure | pancreatic pseudocyst | pancreatic encephalopathy | thrombophlebitis | C | C,C | C,C | 0.7493 |
| Which of the following symptoms in patients with acute pancreatitis is a sign of severe disease and poor prognosis? | metabolic acidosis | metabolic alkalosis | Hypokalemia | hypomagnesemia | hypocalcemia | E | A,A | E,E | 0.6794 |
| The clinical manifestations of acute pancreatitis are incorrectly | Shock is only seen in the hemorrhagic and necrotic type | All had sudden onset of upper abdominal pain | Paralytic intestinal obstruction may occur | Most have mild fever | Acute respiratory distress syndrome may occur | B | C,B | A,A | 0.3809 |
| The clinical manifestations of acute severe pancreatitis generally do not include | shock | respiratory failure | fever | diarrhea | gastrointestinal bleeding | D | D,D | D,E | 0.5315 |
| Which of the following is not a major complication of acute hemorrhagic necrotizing pancreatitis | gastrointestinal bleeding | adult respiratory distress syndrome | acute renal failure | heart failure | pancreatogenic diarrhea | E | E,B | D,E | 0.7429 |
| Which of the following is a characteristic symptom of acute pancreatitis? | Increased serum amylase | stomach ache | pancreatic pseudocyst | Steatorrhea |  | A | A,A | A,B | 0.5835 |
| **Treatment** |  |  |  |  |  |  |  |  |  |
| Which statement is incorrect regarding the treatment of acute pancreatitis? | Fasting and intestinal decompression to reduce acid secretion and pancreatic juice secretion | Octreotide is an effective drug in treating hemorrhagic and necrotizing pancreatitis | When the pain is severe and the effect of atropine is not good, morphine can be added at the same time. | Adrenocorticoids are only indicated for hemorrhagic necrotizing pancreatitis associated with shock or adult respiratory distress syndrome | Intestinal enzyme treatment is only suitable for the early stage of hemorrhagic necrotizing pancreatitis | C | D,C | C,B | 0.8409 |
| When patients with acute pancreatitis develop shock within 2 days of onset, the first treatment measures should be | Intravenous infusion of pectin | Quickly replenish blood volume | intravenous dexamethasone | IV antibiotics | intravenous infusion of alamin | B | B,B | B,B | 0.8628 |
| In the treatment of acute pancreatitis, the following drugs inhibit pancreatic enzyme activity: | pectin | glucagon | calcitonin | somatostatin | Octreot | A | A,A | D,A | 0.5452 |
| The most important reason for fasting in the treatment of acute pancreatitis is | Because the patient is vomiting | Because the patient has abdominal distension | Because the patient has abdominal pain | Reduce gastric acid secretion | Reduce pancreatic juice secretion | E | E,E | E,E | 0.8636 |
| A 45-year-old man developed mid-upper abdominal pain radiating to both waists 8 hours after drinking alcohol, accompanied by nausea and vomiting. For those with difficulty in diagnosis, further measures should be taken | laparotomy | ERCP check | Close observation under anti-infective treatment | anti-shock therapy | Anti-shock and simultaneous abdominal puncture | E | D,D | D,B | 0.3926 |
| Male, 45 years old. After eating a high-fat meal and drinking alcohol, the pain in the upper abdomen continued for 8 hours, and the pain was not relieved after vomiting twice. Check, left upper abdominal tenderness and rebound tenderness: T37.8°C. If antibiotic treatment is required, the best combination of antibiotics is metronidazole and | Azithromycin | Clindamycin | Ciprofloxacin | Imipenem | penicillin | C | C,C | D,C | 0.4253 |
| Male, 41 years old. Upper abdominal pain for 7 hours. She was accompanied by fever with a body temperature of 38.5°C and frequent vomiting. Physical examination revealed upper abdominal muscle tension and tenderness without shifting dullness. White blood cells 15X10°/L. X-ray examination: no free gas was found under the septum. The basic measures of treatment are | emergency surgery | Fasting and gastrointestinal decompression | Abdominal puncture and drainage | Laparoscopic gallbladder removal | Apply large amounts of broad-spectrum antibiotics | B | B,B | B,B | 0.8532 |
| Which of the following is inappropriate for the treatment of acute pancreatitis? | gastrointestinal decompression | Early application of glucocorticoids | Acid suppression therapy | pain relief treatment | none | B | B,B | B,B | 0.7345 |
| Drugs that are more effective in treating acute hemorrhagic and necrotizing pancreatitis | Octreot | adrenal glucocorticoid | H2 receptor antagonists | proton blocker | anticholinergic drugs | A | B,B | A,A | 0.6908 |
| The main measures to treat acute pancreatitis (edematous type) are | Operation | Inhibit or reduce pancreatic secretion | Adrenocorticosteroids | Apply antibiotics | Use morphine for analgesia | B | D,B | B,B | 0.8785 |
| When treating patients with severe pancreatitis combined with intestinal paralysis, the drugs that should not be used are: | antibiotic | antacids | Acid suppressants | anticholinergics | Drugs that inhibit pancreatic enzyme activity | D | D,C | D,D | 0.77 |
| The role of somatostatin in the treatment of hemorrhagic necrotizing pancreatitis is incorrectly described | Inhibits the secretion of pancreatic juice and pancreatic enzymes | Inhibits pancreatic enzyme synthesis | Does not relieve abdominal pain | Reduce local complications | none | C | E,C | B,C | 0.4399 |
| **Etiology** |  |  |  |  |  |  |  |  |  |
| Most common causes of acute pancreatitis | pancreatic duct obstruction | heavy drinking | Biliary tract disease | surgical trauma | Infect | C | C,C | C,C | 0.7257 |
| 65-year-old male was admitted to the hospital with acute pancreatitis and multiple organ dysfunction syndrome. Analyzing the mechanism of its occurrence, it was found that the factors that are not important damaging factors are | Cytokines | inflammatory mediators | growth factors | systemic inflammatory response | tissue ischemia-reperfusion process | C | C,C | C,C | 0.5814 |
| The main cause of acute pancreatitis in China is | chronic alcoholism | Biliary system diseases | Endocrine and metabolic disorders | pancreatic duct obstruction | Surgery and Trauma | B | B,B | B,B | 0.9573 |
| The most common causative factors of acute pancreatitis in Western countries are | Overeating | alcoholism | biliary stone disease | Gastroenteritis | Parathyroid function improved | B | B,B | C,B | 0.9313 |
| The following drugs have been confirmed to induce acute pancreatitis, except which one | Thiazide diuretics | adrenal glucocorticoid | somatostatin | Sulfa drugs | azathioprine | C | C,C | C,C | 0.7809 |
| Pancreatic necrosis is caused by | Activate elastic fiber enzyme | Activate phospholipase A | activate pancreatic vasodilator | Activate trypsin | Activate plasma inflation | D | D,D | D,D | 0.446 |
| In the pathogenesis of acute pancreatitis, the enzyme closely related to blood vessel destruction leading to bleeding is | Lipase | Phospholipase A | Elastase | chymotrypsin | Trypsin | C | C,C | B,B | 0.5035 |
| The enzyme that plays a key role in the pathogenesis of acute pancreatitis is | amylase | Elastase | Trypsin | Kinase |  | C | C,C | C,C | 0.8727 |
| The most common causes of acute pancreatitis in Chinese | drug | Viral infection | Alcohol | Biliary system diseases | self-immune | D | D,D | D,D | 0.9681 |
